# Virtual reconstruction of stone tool refittings by using 3D modelling and the Blender Engine: The application of the “ReViBE” protocol to the archaeological record

**DOI:** 10.1371/journal.pone.0309611

**Published:** 2024-08-29

**Authors:** Javier Sánchez-Martínez, Katia Calmet, Jorge Martínez Moreno, Xavier Roda Gilabert

**Affiliations:** 1 Centre d’Estudis del Patrimoni Arqueològic de la Prehistòria (CEPAP-UAB), Universitat Autònoma de Barcelona, Barcelona, Spain; 2 Interdisciplinary Center for Archaeology and the Evolution of Human Behaviour (ICArEHB), Universidade do Algarve, Faro, Portugal; 3 Departament d’Humanitats, CASEs - Culture, Archaeology, and Socio-Ecological Dynamics Group, Universitat Pompeu Fabra, Barcelona, Spain; Universita degli Studi di Ferrara, ITALY

## Abstract

Visual representation of material culture plays a crucial role in prehistoric archaeology, from academic research to public outreach and communication. Scientific illustration is a valuable tool for visualising lithic artefacts and refittings, where technical attributes must be drawn to enhance our understanding of their significance. However, the representation of lithic refittings, which involve dynamic and sequential transformations of a volume, requires an alternative approach to traditional two-dimensional models such as photography or illustration. Advances in imaging technologies have improved our ability to capture and communicate the multifaceted nature of archaeological artefacts. In this context, we present the ReViBE protocol (Refitting Visualisation using Blender Engine), which integrates photogrammetry, 3D modelling and the animation software Blender© for the virtual representation of lithic refittings. This protocol allows the sequential study of core reduction phases and their associated flakes, as well as other aspects related to knapping decision making (core rotations, surface modifications, and direction and position of impact points). Thus, this method allows the visualisation of techno-cognitive aspects involved in core reduction through a step-by-step animation process. In addition, the 3D models and virtual reconstructions generated by ReViBE can be accessed through open repositories, in line with the principles of open science and FAIR (Findable, Accessible, Interoperable, and Reusable) data. This accessibility ensures that data on lithic technology and human behaviour are widely available, promoting transparency and knowledge sharing, and enabling remote lithic analysis. This in turn breaks down geographical barriers and encourages scientific collaboration.

## 1. Introduction

Stone tools have a wide geographical and chronological distribution and are of considerable cultural value in the archaeological record. They serve as valuable references for understanding the evolutionary history of hominins [1–3] and variations in past human behaviour [4–6].

Uncovering evidence of past human activity, in which stone tools play a critical role [7, 8], is a fundamental goal of anthropological and archaeological research [9, 10]. However, identifying such evidence within the archaeological record can be challenging because site formation processes [11] are intertwined with contextual resolution, potentially leading to misinterpretation and distortion of past human activities.

In this regard, refittings significantly enhance archaeological interpretations, serving as high-resolution temporal units that allow the visualisation of human behaviour within the archaeological record [12–15]. In this perspective, refittings have proven useful for understanding site formation processes from a taphonomic and anthropological perspective, shedding light on the concept of ’internal time’ within archaeological assemblages [16, 17], and refining the contextual resolution of sites [18, 19]. This is especially useful to address diachronic and synchronic stratigraphic relationships in time-average deposits [15, 20, 21], and post-syn depositional site formation processes [22–29]. Additionally, refittings allow the analysis of technical behaviours [30–32], intra-site social organisation [15, 33, 34], site functionality [35–38] and mobility [39, 40].

In this framework, lithic refittings provide insight into the anthropological nature of human occupations contributing to the understanding of intra-site technological processes and spatial relationships. In parallel to this, lithic refittings have been used to understand artefact volumetric reduction, providing a dynamic perspective of the knapping sequence, which is essential for the analysis of techno-cognitive aspects in technological organisation [41, 42]. These elements are relevant for identifying variations in the technological adaptations of past societies (see references in [18, 19]), which have played an important role in defining cultural traditions over a wide spatial and temporal range [43].

Overall, refittings allow researchers to visualise cognitively opaque knowledge [44]. In other words, they allow reconstructing and visualising vast range of actions and technical decisions comprised in the end product (core), which would not be fully visible through observation of each refit piece individually. For this reason, refits are one of the most powerful tools for identifying variations in human behaviour at prehistoric sites [31].

### 1.1. The application of imaging science and technologies to lithic refits

The graphic representation of material culture is central to all areas of science, from basic research to public engagement through events, exhibitions, and museums (for a review see [45]). Scientific illustration serves as a useful tool for the representation of archaeological artefacts, particularly stone tools, which require a detailed representation of their technical attributes for their understanding [4, 46–50]. Traditional drawing remains as one of the main methods of visualising lithics, as shown by the handbooks published in the last two decades [51–53]. However, advances in imaging science and technology offer improvements in the representation of archaeological artefacts, allowing the integration of different types of information depending on the research objectives [54–58]. This is especially important in the case of refits which provide relevant information on the techno-cognitive sphere of past populations. Furthermore, refittings serve as reference elements for the study of technical organisation and human behaviour at prehistoric sites [14, 37, 59–62].

However, although refitting represents a dynamic process involving a variety of artefacts at different phases of volumetric reduction, their scientific representation has been primarily in 2D through illustration or photography (but see [63]). The two-dimensional representation of refittings does not allow for the visualisation of key characteristics of a refitted assemblage (e.g. the number of pieces involved, morphometric variability, artefact attributes or the presence of fractures.). Without identifying these elements, researchers lack visual and technological information about the reduction phase, which limits their analytical capacity. In addition, there are no established guidelines for the visual representation of refits. As a result, the data extracted from them can sometimes be diffuse, limited or insufficient.

In recent years, new forms of graphical representation that combine different techniques, such as three-dimensional (3D) modelling and photogrammetry, have been applied for the visualisation of archaeological objects [64, 65]. 3D models can be generated by scanning the surface of the object using either fixed or portable scanners [58, 66–69]. The most powerful and high-resolution scanners for archaeological objects tend to be stationary, although there are now fast portable scanners that also cover the applications of fixed scanners [70, 71].

Alternatively, photogrammetry allows the creation of 3D models from digital photographs and is a widely used technique in archaeology. It is easy to use and does not require significant financial resources, requiring only a good digital camera and a suitable working environment for model-making [72].

Scientific illustration is useful for depicting technical attributes in individual lithic artefacts (e.g. direction of removal, percussion ripples or retouching). However, archaeological drawing has limitations in depicting refitting sequences because the technical attributes of an artefact may be overlaid by others from previous phases [73]. In some cases, simplified schematics are used to illustrate refitting (e.g. [13, 74, 75]).

On the other hand, photography is a valuable resource as it is accessible, inexpensive and allows the visualisation of textures, colours and different types of materials. It is advisable to represent different views of a refit to obtain a better volumetric perception of the assemblage and to avoid a static perspective [32, 37]. Some protocols, such as STIVA, combine photography and digital drawing, enhancing both methods of representation without requiring a significant time investment [57].

Advances in imaging technologies have expanded the possibilities for representing and analysing archaeological objects (e.g. [34, 76–78]), including the visualisation of refitting assemblages [45] following their reduction sequence [14] or using sequential photography [45]. Given this emergent field, we propose the implementation of the **Re**fitting **Vi**sualisation on **B**lender **E**ngine (ReViBE) protocol, which aims to dynamically visualise refitting sets.

## 2. Methods

One of the limitations of lithic refittings relies on how they are presented to the scientific community (mainly combining 2D methods), which imposes some constraints to assess tecno-cognitive aspects from volumetric reduction sequences. However, these limitations can be overcome by using sequential 3D animation of the refits. The ReViBE protocol described in this article, combines three-dimensional models of archaeological materials with the animation software Blender© to generate animations on lithic refittings. This protocol is published on the digital platform for reproducible methods Protocols.io dx.doi.org/10.17504/protocols.io.ewov1qxqkgr2/v2 and is available as S1 File to this paper. As a reference to illustrate this protocol, we have used the refitting set No. 41 from the Early Upper Palaeolithic unit 497D of the Cova Gran de Santa Linya. However, other refitting sets have been used in the development of the protocol.

ReViBE is conceived to work with refitted assemblages. Independently of the methodology used in the refitting process. The workflow used for the identification of refittings is explained in the study case of this article.

ReViBE uses the 3D models of each lithic artefact that composes the refit as a baseline to work in a digital environment. This process can be carried out by scanning the artefact surfaces (using micro-CT or both portable and fixed scanners, ensuring good or medium resolution) or by photogrammetry. Both methods may have limitations depending on the specific archaeological application in which they are used [58].

**Fig 1:**
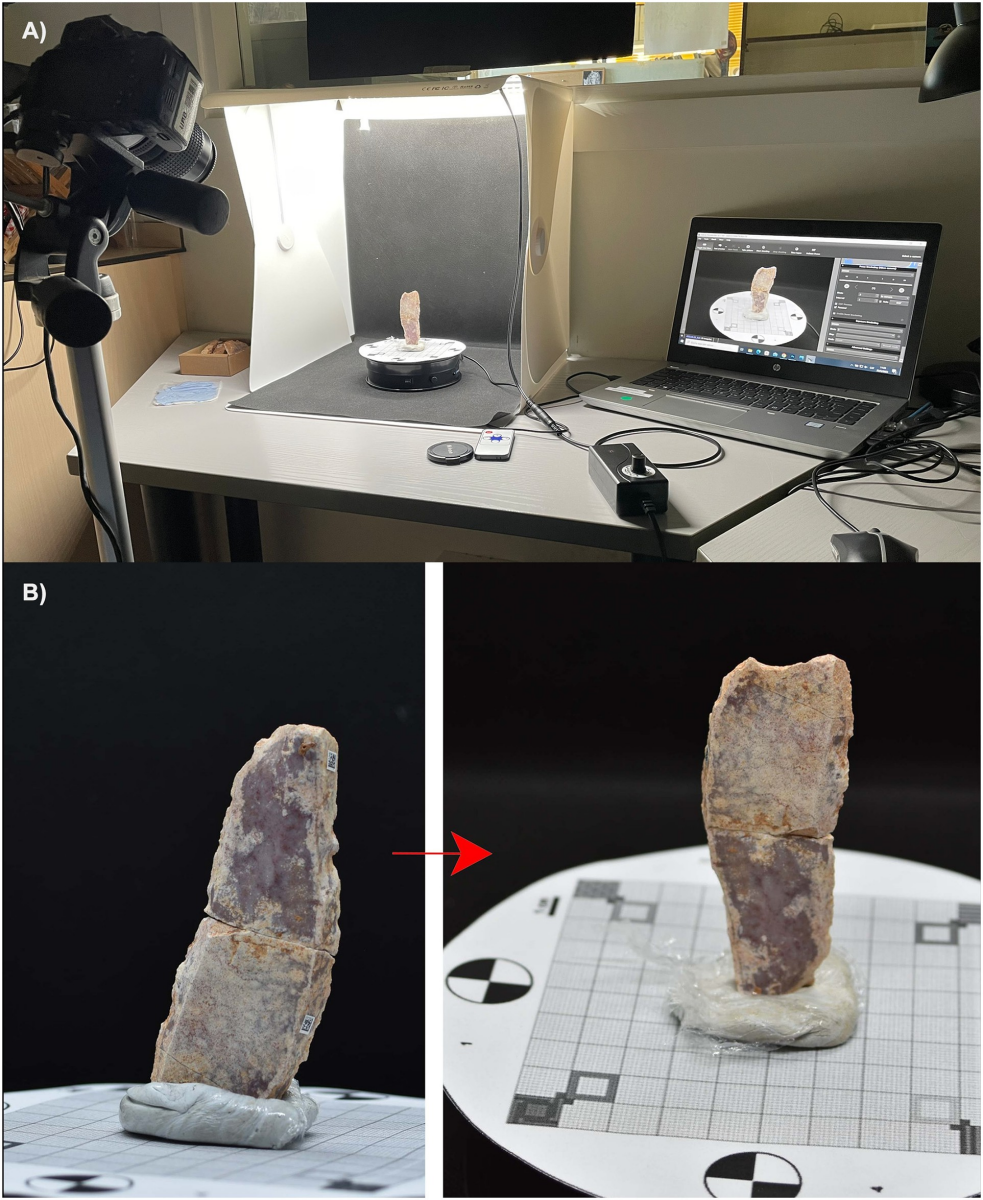
Photogrammetry applied to one of the artefacts of the refitting set No. 41 from level 497D of Cova Gran de Santa Linya. A) Photogrammetry set up with the camera at a high-angle. B) Change of the position of the artefact after the first round of enveloping photographs using frontal and high-angle. The piece will be rotated 180º to capture possible covered surfaces by the adhesive.

In this protocol, we have used photogrammetry to obtain the volumetric information of each artefact and Agisoft PhotoScan © to transform the captured images into a three-dimensional model. This method is widely known [72], easy to perform and affordable, which improves the reproducibility of the protocol. Photogrammetry was conducted by taking a series of enveloping photographs of the archaeological piece (one capture for each 10º rotation) using a frontal and high angle (S1 Part 2, 9–17 in S1 File). After this, the piece was rotated 180º to record the opposite part (Fig 1). A detailed list of the materials and software used in the process can be found in Table 1.

**Table 1:** List of materials and software used along the REVIBE protocol workflow.

Equipment	Function	Description
Camera	Image capture	Ideally with a 35mm–80mm focal length
Tripod	Camera support	
Lightbox	Illumination and background control	Alternatively, three lights can be used to create the basic triangle of lighting and a black velvet fabric for the background
PC or laptop	Data processing	Hardware must meet the minimum system requirements for the selected software solutions
Rotating turntable	Object support	It can be automatic or manual
Modelling clay	Object holder	Reusable adhesive mastic can be an alternative (e.g. *Blue-Tack* or similar products)
Plastic Wrap	Protection of the object from oils and stickiness of the modelling clay	
Photogrammetric scale marker	Scaling up the object	Three free versions of the photogrammetric scale can be downloaded at https://conservancy.umn.edu/handle/11299/172480
Helicon Remote	Remotely control the camera’s shots	Can be use any other control remote software for this task
Agisoft PhotoScan	Create a 3D image from a set of images in 2D	Any other photogrammetric 3D software can be used for this task. See Table 2
Blender	Create the video animation of the reconstruction	Any other 3D animation software can be used for this task. See Table 2

The images, obtained through the detection of common area points (S1 Part 3 in S1 File), are processed by software that converts them into point patterns, creating the 3D image of the object. Additional attributes, such as the original texture of the artefacts, can be incorporated to reconstruct surface conditions during this process. This enables the identification of the raw material of the piece and potential surface modifications, such as patina or burning. Several tools and sources can be used for the conversion of sets of pictures into 3D images. We summarise some of the options in each of the main steps of the protocol, mentioning both free and paid access solutions (Table 2). Alternative software solutions and free applications can be found in [79].

**Table 2:** List of applications available for each of the phases of the data collection process in each of the sections of the protocol.

Step	Tools & Sources
Remote control photo software (for all camera brands)	Public Licence: digiCamControl (https://digicamcontrol.com/)
	Proprietary License: Helicon Remote (https://www.heliconsoft.com/heliconsoft-products/helicon-remote/)
Converting images to 3D	Public Licence: MeshLab (https://www.meshlab.net/) Meshmixxer (https://meshmixer.com/) Meshroom (https://alicevision.org/#meshroom/) 3DFZephyr (https://www.3dflow.net/3df-zephyr-photogrammetry-software/) PhotoCatch (iOS) https://apps.apple.com/es/app/photocatch/id1576081762) Polycam (App) https://poly.cam/) 3D Scanner (Iphone App) https://apps.apple.com/es/app/3d-scanner-app/id1419913995) EyesCloud 3D (https://www.eyescloud3d.com/login) Widar (App) (https://www.widar.io/) Qlone (App) (https://www.qlone.pro/download) Kiri (App) (https://www.kiriengine.com/) Trnio (App) (https://www.trnio.com/product-page)
	Proprietary License: Photoscan/Metashape (https://www.geobit.es/producto/agisoft-photoscan-profesional/) Reality capture (https://www.capturingreality.com/realitycapture)
Creating animation sequences	Public Licence: Blender (https://www.blender.org/) Houdini (https://www.sidefx.com/products/houdini-apprentice/) Unreal Engine (https://www.unrealengine.com/en-US/download)
	Proprietary License: Cinema 4D (https://www.maxon.net/es/cinema-4d) Maya Autodesk (https://www.autodesk.es/products/maya/overview?term=1-YEAR&tab=subscription) Zbrush (https://www.maxon.net/es/zbrush) Unity (https://unity.com/es)
Repositories	Public Open repositories: Zenodo (http://zenodo.org/) FigShare (http://figshare.com) Mendeley Data (https://data.mendeley.com/) DataHub (http://datahub.io)
	Institutional Open repositories:Platforms governed by national or transnational regulations to deposit documents in open access. Based in protocols such as: CORA (https://cora.csuc.cat/es/) Catalan Open Research Area OpenAIRE compliant (https://www.openaire.eu/) adapted to the metadata requirements of the EU Open Archive Initiative-Protocol for Metadata Harvesting (OAI-PMH) (https://www.openarchives.org/) National Science Foundation (NSF,US)

Once the three-dimensional models of each artefact have been created, they are uploaded into Blender to animate the refit sequence (S1 Part 4 in S1 File). The animation is composed of three main variables: a) the individual artefacts that form the refit; b) the motions assigned to each artefact within the reduction sequence; c) the time applied between the motions of each artefact and the refitting.

To create the movements along the knapping sequence, we work in individual timelines for each piece. The use of keyframes, which can be activated using the tool of the same name, allows us to record the start and end of each movement action. From here, the software generates the intermediate movements to achieve the desired flow. In this way, each artefact can be controlled separately and sequentially within the reduction sequence.

**Fig 2:**
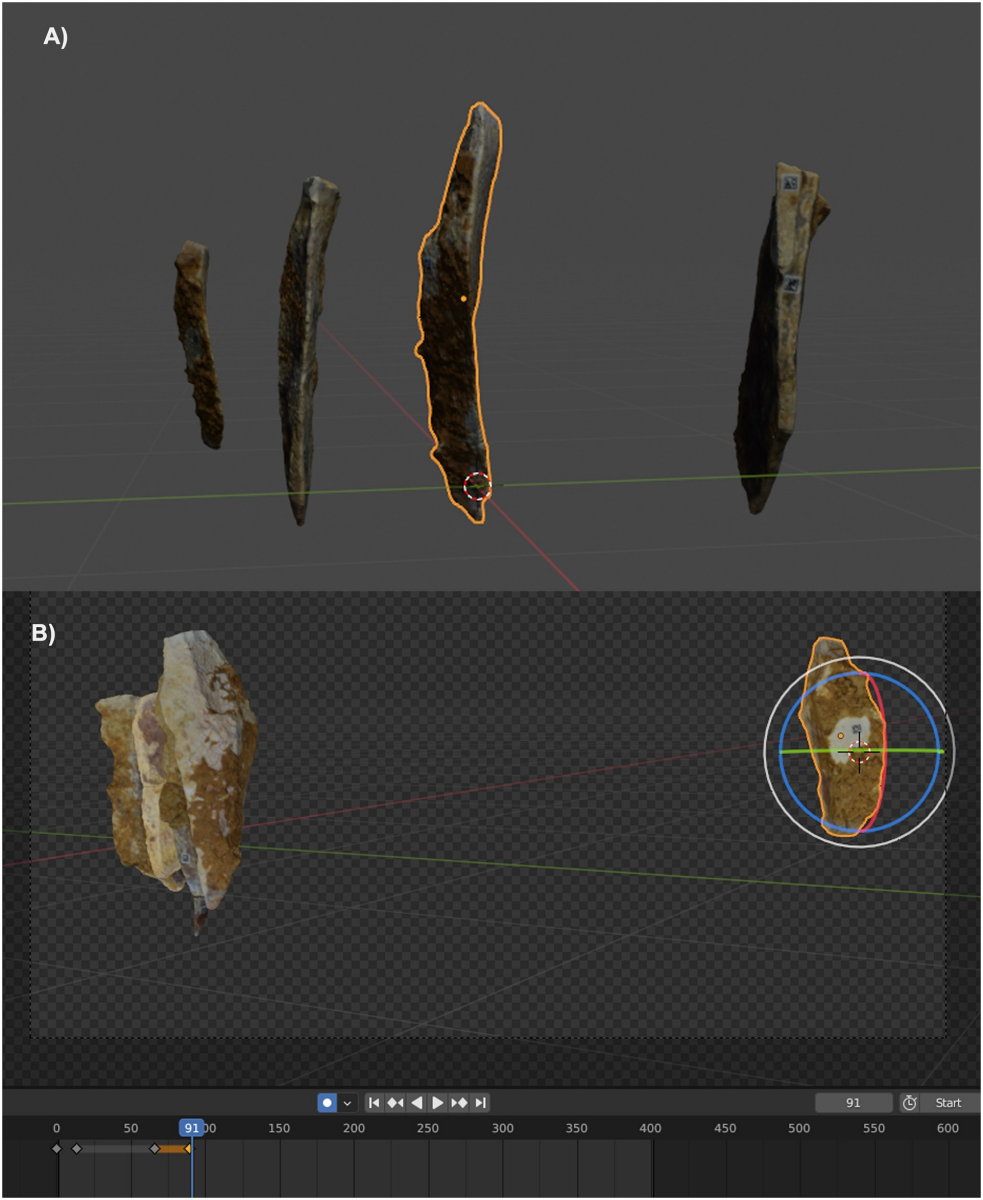
Virtual reconstruction process of the knapping sequence of the refitting No. 41 on *Blender ©*. A) Seting up of the motion of each object in the workspace by selecting a central mass point B) Rotation motion applied to one of the artefacts of the refitting. The motion must be associated with a specific time frame to create the animation. At the bottom: Timeline view with keyframes per motion action.

Both, the motion, and the time variable can be modified to highlight some of the technical processes involved in the refit (e. g. the abandonment of a knapping surface and the opening of a new one, the rejuvenation of a knapping platform, or the core maintenance). To highlight these processes along with the animation, some graphical solutions can be displayed. In this protocol, the movement that the hammerstone would follow during the reduction phase, is represented by an arrow, allowing the rhythm and orientation of the knapping process to be identified (Fig 2). The arrow also indicates where the hammerstone impacts the core and where lithic blanks are removed from the original volume. To insert this type of element in the animation, we can use a 2D video editing software, in this case the open-source *Davinci Resolve ©*. The same keyframe animation process described above will be applied to these shapes.

The animations created by *Blender ©* allow the representation various aspects of the refitted reduction sequence, being a flexible tool to reconstruct knapping processes (e.g. different reduction phases, the abandonment of core platforms, or core rotations among others). In this way, each of the actions carried out during the reduction is shown in consecutive and sequential order, enhancing the understanding of the production and maintenance phases within a refit.

The software also allows the camera to move in a three-dimensional workspace, enabling it to highlight technological features by zooming in at any point in the sequence. Technical and technological attributes on the pieces can be represented by adapting the symbology from archaeological drawings and inserting them throughout the animation or after the movement action of each piece has been completed [52, 53].

Although 3D animation programs in general, and *Blender ©* in particular, require use experience, the application of a step-by-step protocol facilitates a standardisation in the use of this type of program in the transfer of knowledge within the field of archaeology.

## 3. Case study

The 3D reconstruction of refittings aims to overcome some of the limitations proposed in the representation of lithic artefacts [14, 55, 63, 80], whose volume is key for understanding lithic reduction and techno-cognitive procedures during knapping [81]. However, understanding lithic reduction not only requires detecting variations in the volume of the object, but also observing how and when the mass was lost from the original volume, and through which decisions and technical actions.

The characterization of knapping methods can be a useful indicator for distinguishing technical trends between Neanderthals and anatomically modern humans, thus helping to understand the complex scenario of the Middle to Upper Palaeolithic Transition. Unit 497D from Cova Gran de Santa Linya is associated with these issues.

This unit has been extensively studied in recent years, providing valuable data on contextual and chronometric information, site formation processes, spatial analysis, lithic technology, and subsistence strategies [14, 21, 82–85]. These studies have provided a comprehensive discussion of transitional technocomplexes and have significantly contributed to expanding our knowledge about the interactions between the last Neanderthals and the first anatomically modern humans in southwestern Europe.

In this respect, the refits provided new data on the technological organization of the human groups living at Cova Gran around 38-40k.cal BP., extending the previous information obtained from the technological analysis of the lithic assemblage [84]. The reconstructions in unit 497D were carried out by following technological analysis and raw material characterisation of the lithic assemblages. All artefacts were categorized based on technical attributes [4–6, 86] and catalogued using Raw Material Unit (RMU) reconstruction [87], adapted to the petrographic characteristics and variability of local chalcedony [88]. This methodology proved successful in achieving refittings, particularly given the challenge posed by internal colour variations in local chalcedony for the identification of lithic connections. This process has led to the identification and technological study of various lithic refits, including long knapping sequences and their respective cores, that were published in previous works [14, 21]. In one of these works [14], animated refittings can be found in the S1 File section.

The refits revealed the technological actions used in the reduction of cores, the production goals and the morphometric characteristics of the lithic blanks. The technical analysis of the refits showed that flakes, blades, and bladelets were obtained interspersed in the same knapping sequence, indicating that bladelet production was not exclusive to bladelet cores. This feature is slightly similar to the one proposed for the Proto-Aurignacian, on which blades and bladelets are obtained from the same core following a single and continuous knapping sequence [89–93] and differs from the Early Aurignacian, where it is proposed that the production of blades and bladelets follows separated reduction schemes [94]. However, recent studies on the Proto-Aurignacian site of Fumane revealed a reduction scheme exclusively oriented to bladelet production, and another one where blades and bladelets are obtained together within the same reduction sequence [81, 92], as occurred in study case from Cova Gran. These new discussions based on technological data are of relevance for the chrono-cultural attribution of 497D within the Middle to Upper Palaeolithic Transition (MUPT) and the Early Upper Palaeolithic [14].

The visualisation and volumetric graphic representation of these refits was crucial in demonstrating the technical aspects of the lithic production. However, long refitting sequences often involve overlapping artefacts, which means that technological information from later phases is obscured by the most recent ones. To address this problem, the digitisation and animation of the refittings were necessary to provide a streamlined approach to their dynamic representation. This process allows the refits to be structured in a temporal sequence, highlighting the characteristics of the blanks and their position within the volumetric reduction.

To incorporate the temporal variable into the reconstruction of volumetric reduction in lithic refits, we used ReViBE. This protocol was applied to the refits to illustrate the technological and chrono-cultural discussion of unit 497D [14]. The animated videos produced by this protocol can be found in the Supplement to the above-mentioned article.

Among the different sets of refits from the 497D unit, refit No. 41 was used as a reference to carry out the current protocol with the step-by-step process [95]. This refit consists of nine pieces that form four morphometrically diverse artefacts, ranging from flakes to elongated blanks.

We have created a flowchart depicting the artefacts involved in refit No. 41 and their temporal relationships in the reduction sequence to document their sequential order. Additionally, technical actions that occur during the reduction, such as platform rejuvenation and changes to the knapping surface, can be noted and incorporated into the flowchart (as convenience), which could be useful in constructing the animation sequences using Blender. This facultative step helps to understand the temporal and technological dimensions of refittings. The first allows the pieces to be placed in a logical and correct order within the reduction sequence; while the second allows the technical actions involved in the management of the core or the detachment of the lithic blanks to be understood.

In refitting No. 41, the knapping sequence occurs on a single surface through the frontal debitage of the volume, indicating that the core was not rotated during the reduction process. The absence of rotation, typically used to create lateral convexity in the core, can lead to the production of morphometrically diverse blanks, which is a notable feature in MUPT assemblages and has been observed in other animated refittings in 497D [14].

## 4. Discussion

The protocol has multiple applications from scientific research to science communication. ReViBE’s contributions to the scientific community are significant and are in line with current needs in archaeological research and the valorisation of material culture and prehistoric heritage. This section outlines the advances of this protocol in: 1) the application of new methodologies to visualise lithic refittings; 2) the interpretation of the archaeological record and archaeologically-based hypothesis; 3) the promotion and study of digital heritage; 4) the development of outreach activities and knowledge transmission strategies; 5) the fostering of remote research.

### 4.1. Methodological advances

The visualisation of assemblages has traditionally been limited to two-dimensional methods, such as archaeological drawing and photography. These “static” methods have limitations in observing sequential processes, such as the volumetric reduction of a core or the pieces obtained during the knapping sequence.

However, the ReViBE protocol offers a solution to this problem by reconstructing the stages of the volumetric reduction through the individual stages of the artefacts within a specific temporal sequence. In other words, it reproduces the stages of volumetric reduction in a continuous temporal sequence without interruptions and individually presents the pieces involved in the refit in a dynamic visual representation. By controlling the spatiotemporal path of each artefact, the reduction sequence is constructed to avoid overlapping technical attributes, core negatives and knapping products. This is particularly significant in hierarchical and/or recurrent knapping strategies, where the lithic production from earlier phases is often covered by later phases. This allows the observation of the technical and morphometric characteristics of the artefacts, their order within the knapping sequence, and their role in the production or maintenance phases to be observed. Furthermore, the temporal frame assigned to each artefact enables controlling how quickly they move in the reduction sequence. This can be useful to highlight technical processes within the reduction. As a result, ReViBE allows the animation of three-dimensional representations, giving more dynamism to knapping processes and avoiding the overlapping of information that occurs in refit sequences. This represents a methodological advancement over current refit visualisation methods.

### 4.2. Archaeological application

The visualisation of reduction sequences in real-time, helps to understand production goals or standardisation of lithics, as well as to evaluate the relevance of knapping sequences in anthropological and organisational terms. The following case concerns the refits found in level 497D of the Cova Gran de Santa Linya [14, 21] and how these remains provide insight into the characteristics of the lithic production. The technological study revealed a lack of correspondence between the bladelets and the laminar cores, whose presence was quite limited. The analysis of the refits showed that bladelets were produced alongside flakes, laminar flakes, and blades in the same reduction sequence. This suggests that the laminar production of the assemblage was not exclusively associated with laminar cores but was integrated into other reduction sequences. This feature is not fully consistent with the characteristics proposed for the Proto-Aurignacian and Early Aurignacian [89, 92, 94, 96] and allows introducing alternative scenarios for the chrono-cultural attribution of this unit. The animation of reconstructed sequences of this unit can now represent the archaeological evidence beyond this debate. This serves as a tool to support hypothesis testing and scientific research.

In parallel to this, ReViBE includes texture on the virtual animation of refittings, which helps to visualise differential patina formation along the refitted artefacts, indicating variations in surface modification across the lithic assemblage due to site-formation processes.

### 4.3. Digital heritage

The study of stone tools has a long tradition in prehistoric archaeology. Among other things, experimental knapping has played a key role in understanding the technology used by past populations, and in making Prehistory known and popular to different audiences. Until now, stone tool making has been presented based on experimental demonstration, in which highly experienced people reproduce the methods used in the past (also based on ethnographic references). This protocol allows the reconstruction of past knapping processes using archaeological material, which is a relevant contribution to the field. This dynamic approach to the visualisation of archaeological data is essential both to strengthen the preservation of the material culture and to promote the digital heritage [97–101]. In the case of stone tools, 3D reconstructions can preserve the pieces from residues and glues used in their study, as well as reducing the micro-chipping and edge breakage caused by the refitting process [55, 63], which is beneficial for use-wear analysis. Scanning large stone-tool assemblages is now more feasible than it was years ago [58], increasing the possibility of conducting virtual refittings without physically touching the pieces [55, 80]. One of the future directions worth exploring involves the use of computational methods to systematise the virtual refitting process by using reference points to detect common areas among the artefacts. This approach could be tested with experimental collections that allow the reconstruction of all stages of the reduction process. Following this, if lithic assemblages are scanned in entirely, lithic refittings could be conducted directly in a virtual environment by matching surface and angle associations between artefacts. However, it is still unknown how to address specific limitations related to the formation of the assemblages and the site, as well as the accuracy of the method with fragments or in assemblages were retouched tools are abundant.

### 4.4. Outreach and knowledge transfer

In recent years, open science and public engagement strategies have proven to be effective in achieving broad science communication. Open science is based on the principles of providing unrestricted access to information and increasing transparency throughout the conceptualisation, development and implementation of research [102]. For this reason, there is a commitment to ensure that knowledge dissemination and science communication adhere to the principles of collaboration, transparency, and accessibility across different formats and audiences, especially when research has been funded by public resources. Open science is a priority for the European Union to improve the quality and effectiveness of research resources, which is one of the key initiatives outlined in Horizon Europe.

In this new context of global scientific awareness, FAIR principles (making data Findable, Accessible, Interoperable, and Reusable) have contributed to make scientific data something to care about, becoming the standard for the dissemination of knowledge from EU-funded scientific research [103, 104]. FAIR principles provide an opportunity to achieve a global understanding of the research environment at institutional, national and international levels and to increase the reliability of research results. In addition, it further strengthens the use of digital and collaborative technologies and promotes the reproducibility of scientific results [105, 106].

Scientific dissemination and knowledge transfer must be integrated into archaeological research in an intersectional and transversal way. The dissemination of results in academic (conferences, workshops) and non-academic (exhibitions, fairs, schools) contexts implies the creation of scientific content and its adaptation to diverse types of audiences. The ReViBE protocol has significant potential to promote the public dimension of digital content creation for use in public outreach and education activities, thus enriching the link between researchers and public institutions. In addition, flexible audiovisual content of archaeological remains and refittings can be used to explain complex technological processes in a dynamic and simple way. In this way, progress can be made in understanding human behaviour and preserving world heritage in digital environments.

### 4.5. Remote research and logistics

The use of digital environments to store archaeological data has increased in recent years. Digital resources are instantly accessible, do not require travel costs and are easy to consult as they are open access [107]. Furthermore, they can overcome some of the limitations of host institutions in terms of consultation due to scheduling and artefact availability. In this respect, ReViBE provides digital content to be consulted in open repositories and proposes new alternatives to work with remote data aligning the commitments of public administrations to open access policies.

In addition, this protocol does not require extensive infrastructure or equipment to operate (Table 1) which is an advantage for setting up under different conditions. 3D models can be created using a digital camera, a common tool in research laboratories, or a scanner. Fixed scanners usually have better resolution, but they tend to be expensive and non-portable. Recently, portable scanners have gained popularity due to their ability to produce convincing results with good resolution [58]. The portability of digital cameras and scanners is an advantage for documenting tasks during fieldwork, allowing the models to be created on the archaeological material using basic infrastructure (e.g. room, lightbox, and tripod), especially in places where there are restrictions on the movement of materials. The basis of ReViBE is also not restricted to any specific software or 3D-capture technique. Although we describe one way of animating refitting, there is a wide range of software options available for each stage of the protocol, including both free and proprietary software (Table 2). This facilitates communication between different target audiences, particularly between researchers and to the public.

## 5. Conclusions

Imaging technologies have recently expanded their applications in archaeological research, transforming the way we document and visualise the archaeological record. The ReViBE protocol includes a new method for visualising refittings by combining photogrammetry, 3D modelling and Blender animation software. ReViBE combines technological and behavioural information from lithic refittings to create virtual content that can be used at different levels. This protocol is publicly available at protocols.io dx.doi.org/10.17504/protocols.io.ewov1qxqkgr2/v2 where the step-by-step workflow for reconstructing refittings in a virtual environment can be followed.

The ReViBE protocol is designed to have an impact across different scientific disciplines and fields. Among other things, this procedure provides a new methodological framework for visualising refittings and lithic blanks in a sequential and temporal order. It introduces movement into the refitting that cannot be conveyed by “static” 2D traditional techniques, thereby increasing the visibility of techno-cognitive aspects in the archaeological record. This methodological advance allows for the dynamic representation of volumetric and temporal information of lithic refitting, which is relevant for understanding archaeologically based hypotheses related to knapping methods, core reduction and lithic production [95, 107]. The archaeological application of this protocol was carried out on several sets of refittings from the MUPT unit of Cova Gran de Santa Linya, and serves to characterise and represent the interspersed production of bladelets in non-bladelet cores. In addition, ReViBE allows the reproduction of knapping sequences using original archaeological artefacts, providing an alternative to knapping demonstration and enhancing the preservation and promotion of the cultural heritage. In parallel to this, ReViBE is a powerful tool for scientific research, outreach and science communication. Virtual content can be disseminated in academic and non-academic environments, enhancing public engagement between different institutions and promoting interoperable and remote analysis among colleagues. This protocol aims to facilitate the conceptualisation of research as a more accessible and transferable resource, contributing to the creation of new scientific content in line with the current Open Science guidelines and the FAIR principles.

In conclusion, ReViBE represents an innovative, accessible, and affordable methodology that can be easily implemented in both research and outreach activities, offering alternatives for the visualisation of material culture. These advances are relevant to the development of prehistoric archaeology and science communication strategies, as well as to the conservation of the cultural heritage in a more interconnected world.

## Supporting information

S1 File The protocol included on this peer-review article is published on protocols.io, (dx.doi.org/10.17504/protocols.io.ewov1qxqkgr2/v2) and is available to download it [94]. The references to the protocol on this article are expressed as (S1 Part X in S1 File). All the raw data needed to replicate this protocol has been uploaded in the Research Data Repository (CORA) (https://doi.org/10.34810/data924), including the pictures taken and used on this protocol (.jpg), the three dimensional model of each refitted artefact (.mtl and.obj), an interactive 3D model of the refitted sequence (3D.pdf) the flowchart of the refitted sequence, the table with technical attributes [108]. A step-by-step explanatory video of this protocol is available in the digital deposit of documents of Universitat Autònoma de Barcelona [107].
(PDF)

## References

[1] Gibson KR, Ingold T. Tools, language and cognition in human evolution: Cambridge University Press; 1993.

[2] Stout D. Stone toolmaking and the evolution of human culture and cognition. Philosophical Transactions of the Royal Society B: Biological Sciences. 2011;366(1567):1050–9. doi: 10.1098/rstb.2010.0369 21357227 PMC3049103

[3] Skibo JM, Schiffer M. People and things: A behavioral approach to material culture: Springer Science & Business Media; 2008.

[4] Inizan ML, Reduron M, Roche H, Tixier J. Technologie de la pierre taillée [Cut stone technology]: Meudon, CREP, CNRS, University of Paris X Nanterre; 1995.

[5] Pelegrin J, Roche H. Evolution et cognition: le temoignage des pierres taillées. Journée Scientifique de l’Association pour la Recherche Cognitive [Internet]. 2000.

[6] Andrefsky JW. Lithic Technology: Measures of Production, Use and Curation. Cambridge: Cambridge University Press; 2008.

[7] de Beaune S. L’homme et l’outil. L’invention technique durant la Préhistoire: CNRS; 2009.

[8] Hussain ST, Soressi M. The technological condition of human evolution: Lithic studies as basic science. Journal of Paleolithic Archaeology. 2021;4(3):25. doi: 10.1007/s41982-021-00098-1 34805748 PMC8591788

[9] Binford LR. In pursuit of the past: decoding the archaeological record: Thames and Hudson, London; 1982.

[10] Schiffer M, Skibo JM, Griffitts J, Hollenback KL, Longacre WA. Behavioral archaeology and the study of technology. American Antiquity. 2001;66(4):729–37. doi: 10.2307/2694186

[11] Schiffer M. Formation processes of the archaeological record. Albuquerque: University of New Mexico Press; 1987.

[12] Karlin C, Bodu P, Ploux S, editors. Who’s who? The Magdalenian flint knapper of Pincevent (France). The Big Puzzle, International Symposium of Refitting Stone Artefacts, Studies in Moderne Archaeology I, Monrepos, Neuwied; 1990: Bonn, Holos.

[13] Delagnes A, Roche H. Late Pliocene hominid knapping skills: the case of Lokalalei 2C, West Turkana, Kenya. Journal of Human Evolution. 2005;48(5):435–72. doi: 10.1016/j.jhevol.2004.12.005 15857650

[14] Martínez-Moreno J, Mora Torcal R, Benito-Calvo A, Roy Sunyer M, Sánchez-Martínez J. A bunch of refits: 497D blade knapping assemblage of the Early Upper Paleolithic in Cova Gran (Northeast Iberia). Archaeological & Anthropological Sciences. 2019;11(9):4585–600.

[15] Vaquero M, Romagnoli F, Bargalló A, Chacón MG, Gómez de Soler B, Picin A, et al. Lithic refitting and intrasite artifact transport: a view from the Middle Paleolithic. Archaeological & Anthropological Sciences. 2019;11:4491–513. doi: 10.1007/s12520-019-00832-5

[16] Bailey G. Time perspectives, palimpsests and the archaeology of time. Journal of Anthropological Archaeology. 2007;26(2):198–223. doi: 10.1016/j.jaa.2006.08.002

[17] Holdaway S, Wandsnider LA. Time in archaeology: an introduction. In: Holdaway S, Wandsnider L, editors. Time In Archaeology: Time Perspectivism Revisited. 1. Salt Lake City: University of Utah Press; 2008. p. 1–12.

[18] Cziesla E, editor The Big Puzzle: international symposium on refitting stone artefacts, Studies in Modern Archaeology. The Big Puzzle, International Symposium of Refitting Stone Artefacts, Studies in Moderne Archaeology I, Monrepos, Neuwied; 1990: Bonn, Holos.

[19] Romagnoli F, Vaquero M. Special Issue in Big Puzzle 30 years after: a multidisciplinary, Paleolithic perspective. Archaeological & Anthropological Sciences. 2019;11(9).

[20] Malinsky-Buller A, Hovers E, Marder O. Making time:‘Living floors’,‘palimpsests’ and site formation processes–A perspective from the open-air Lower Paleolithic site of Revadim Quarry, Israel. Journal of Anthropological Archaeology. 2011;30(2):89–101. doi: 10.1016/j.jaa.2010.11.002

[21] Mora Torcal R, Roy Sunyer M, Martínez-Moreno J, Benito-Calvo A, Samper Carro S. Inside the palimpsest: identifying short occupations in the 497D level of Cova Gran (Iberia). In: Cascalheira J, Picin A, editors. Short-Term Occupations in Paleolithic Archaeology: Definition and Interpretation: Springer; 2020. p. 39–69.

[22] Villa P. Conjoinable pieces and site formation processes. American Antiquity. 1982;47(2):276–90. doi: 10.2307/279901

[23] Hofman JL, Enloe JG. Piecing together the past: applications of refitting studies in archaeology: BAR Publishing; 1992.

[24] Sisk L, Shea JJ. Intrasite spatial variation of the Omo Kibish Middle Stone Age assemblages: artifact refitting and distribution patterns. Journal of Human Evolution. 2008;55(3):486–500. doi: 10.1016/j.jhevol.2008.05.016 18692863

[25] Rosell J, Blasco R, Fernández-Laso MC, Vaquero M, Carbonell E. Connecting areas: faunal refits as a diagnostic element to identify synchronicity in the Abric Romaní archaeological assemblages. Quaternary International. 2012;252:56–67. doi: 10.1016/j.quaint.2011.02.019

[26] Gabucio MJ, Cáceres I, Rivals F, Bargalló A, Rosell J, Saladié P, et al. Unraveling a Neanderthal palimpsest from a zooarcheological and taphonomic perspective. Archaeological & Anthropological Sciences. 2018;10:197–222. doi: 10.1007/s12520-016-0343-y

[27] Vaquero M, Fernández-Laso MC, Chacón MG, Romagnoli F, Rosell J, Sañudo P. Moving things: comparing lithic and bone refits from a Middle Paleolithic site. Journal of Anthropological Archaeology. 2017;48:262–80. doi: 10.1016/j.jaa.2017.09.001

[28] Deschamps M, Zilhão J. Assessing site formation and assemblage integrity through stone tool refitting at Gruta da Oliveira (Almonda karst system, Torres Novas, Portugal): A Middle Paleolithic case study. Plos One. 2018;13(2):e0192423. doi: 10.1371/journal.pone.0192423 29451892 PMC5815586

[29] López-Ortega E, Rodríguez-Álvarez X-P, Ollé A, Lozano S. Lithic refits as a tool to reinforce postdepositional analysis. Archaeological & Anthropological Sciences. 2019;11:4555–68. doi: 10.1007/s12520-019-00808-5

[30] Aubry T, Barbosa AF, Gameiro C, Luís L, Matias H, Santos A, et al. De regresso à Cardina, 13 anos depois: resultados preliminares dos trabalhos arqueológicos de 2014 no Vale do Côa. Revista Portuguesa de Arqueologia. 2015;18:5–26.

[31] Romagnoli F, Vaquero M. The challenges of applying refitting analysis in the Palaeolithic archaeology of the twenty-first century: an actualised overview and future perspectives. Archaeological & Anthropological Sciences. 2019;11:4387–96. doi: 10.1007/s12520-019-00888-3

[32] Vandendriessche H, Crombé P. Formalized Reduction Sequences from the Site of Kerkhove, Belgium–New Perspectives on Early Mesolithic Flint Knapping. Lithic Technology. 2020;45(2):110–24. doi: 10.1080/01977261.2020.1721162

[33] Cattin M-I. El remuntatge de les restes lítiques: organització interna dels assentaments i lligams entre jaciments. Cota zero: revista d’arqueologia i ciència. 2002;11:117–28.

[34] de la Torre I, Martínez-Moreno J, Mora R. When bones are not enough: Lithic refits and occupation dynamics in the Middle Palaeolithic level 10 of Roca dels Bous (Catalunya, Spain). In: Seetah K, Gravina B, editors. Bones for tools—tools for bones The interplay between objects and objectives. Cambridge: McDonald Institute for Archaeological Research; 2012. p. 13–23.

[35] Julien M, Audouze F, Baffier D, Bodu P, Coudret P, David F, et al., editors. Organisation de l’espace et fonction des habitats magdaléniens du Bassin parisien. De la Loire à l’Oder: les civilisations du Paléolithique final dans le nord‑ouest européen: colloque UISPP, commission 8, Liège 1985; 1988: BAR International Series.

[36] Olive M. Une habitation magdalénienne d’Etiolles: l’unité P 151988.

[37] Karlin C, Julien M. An autumn at Pincevent (Seine-et-Marne, France): refitting for an ethnographic approach of a Magdalenian settlement. Archaeological & Anthropological Sciences. 2019;11(9):4437–65.. doi: 10.1007/s12520-019-00860-1

[38] Cattin M-I. Parcours de burins, de la fabrication au rejet: exemples issus des sites magdaléniens de Champréveyres et Monruz (Suisse). In: de Araújo Igreja MB, J-P;Le Brun-Ricalens, F., editor. Burins préhistoriques: formes, fonctionnements, fonctions: Luxembourg: Musée National d’Histoire et d’Art; 2006. p. 241–54.

[39] Close A. Reconstructing movement in prehistory. Journal of Archaeological Method and Theory. 2000;7(1):49–77. doi: 10.1023/A:1009560628428

[40] Taller A, Kieselbach P, Conard NJ. Reconstructing technology, mobility and land use via intra-and inter-site refits from the Gravettian of the Swabian Jura. Archaeological & Anthropological Sciences. 2019;11:4423–35. doi: 10.1007/s12520-019-00778-8

[41] Boëda E. Techno-logique & technologie. Une Paléo-histoire des objets lithiques tranchants: Paris:@ rchéo-éditions. com.; 2013.

[42] Stout D, Khreisheh N. Skill learning and human brain evolution: An experimental approach. Cambridge Archaeological Journal. 2015;25(4):867–75. doi: 10.1017/S0959774315000359

[43] Reynolds N, Riede F. House of cards: cultural taxonomy and the study of the European Upper Palaeolithic. Antiquity. 2019;93(371):1350–8. doi: 10.15184/aqy.2019.49

[44] Csibra G, Gergely G. Natural pedagogy as evolutionary adaptation. Philosophical Transactions of the Royal Society B: Biological Sciences. 2011;366(1567):1149–57. doi: 10.1098/rstb.2010.0319 21357237 PMC3049090

[45] Hussain ST. On the epistemology of stone artefact analysis. In: de Beaune SA, Guidi A, Moro Abadía O, Tarantini M, editors. New Advances in the History of Archaeology: Proceedings of the XVIII UISPP World Congress (4–9 June 2018, Paris, France) Volume 16 (Sessions Organised by the History of Archaeology Scientific Commission at the XVIII World UISPP): Archaeopress Publishing Ltd; 2021. p. 138–70.

[46] Dauvois M. Précis de dessin dynamique et structural des industries lithiques préhistoriques: Pierre Fanlac; 1976.

[47] Laurent P. Le dessin des objets préhistoriques: une introduction/Drawing of prehistoric implements: an introduction. Revue archéologique du Centre de la France. 1985;24(1):83–96. doi: 10.3406/racf.1985.2423

[48] Martingell H, Saville A. The illustration of lithic artefacts: a guide to drawing stone tools for specialist reports: Association of Archaeological Illustrators & Surveyors and the Lithics; 1988.

[49] Adkins L, Adkins R. Archaeological illustration: Cambridge University Press; 1989.

[50] Assié Y. Dessin de l’industrie lithique préhistorique: Notions élémentaires et conseils pratiques. Préhistoire Anthropologie Méditerranéennes. 1995;4:191–227.

[51] Steiner M, Allason-Jones L. Approaches to archaeological illustration: a handbook. York: Council for British Archaeology; 2005.

[52] Raczynski-Henk Y. Drawing lithic artefacts: Sidestone Press; 2017.

[53] Fernández de la Peña J, Castañeda Clemente N. Dibujando el pasado. Una historia de la documentación gráfica en el patrimonio arqueológico: Laergasula Ediciones; 2022.

[54] Rubio Gil D, Martínez Rubio J, Baena Preysler J, Fernández Martín JJ, Codes J. Nuevos métodos para viejas tecnologías: análisis y documentación de los materiales arqueológicos mediante la aplicación de sistemas Láser-scanner 3D. Virtual Archaeology Review. 2010;1(1):169–73.

[55] Delpiano D, Cocilova A, Zangrossi F, Peresani M. Potentialities of the virtual analysis of lithic refitting: case studies from the Middle and Upper Paleolithic. Archaeological & Anthropological Sciences. 2019;11:4467–89. doi: 10.1007/s12520-019-00779-7

[56] Cauche D. Le dessin scientifique des outils lithiques préhistoriques et ses normes. Exemple des collections de la grotte de l’Observatoire (Monaco). Bulletin du Musée d’Anthropologie préhistorique de Monaco. 2020;59:29–40.

[57] Cerasoni JN. Vectorial application for the illustration of archaeological lithic artefacts using the “Stone Tools Illustrations with Vector Art”(STIVA) Method. Plos One. 2021;16(5):e0251466. doi: 10.1371/journal.pone.0251466 33975331 PMC8112888

[58] Göldner D, Karakostis FA, Falcucci A. Practical and technical aspects for the 3D scanning of lithic artefacts using micro-computed tomography techniques and laser light scanners for subsequent geometric morphometric analysis. Introducing the StyroStone protocol. Plos One. 2022;17(4):e0267163. doi: 10.1371/journal.pone.0267163 35446900 PMC9022823

[59] Le Brun-Ricalens F, Brou L. Burins carénés-nucléus à lamelles: identification d’une chaîne opératoire particulière à Thèmes (Yonne) et implications. Bulletin de la Société préhistorique française. 2003:67–83. doi: 10.3406/bspf.2003.12793

[60] de Araújo Igreja M, Bracco J, Le Brun-Ricalens F, editors. Burins préhistoriques: formes, fonctionnements, fonctions. Actes de la Table Ronde international d’Aix-en-Provence 2006: Luxembourg: MuséeNationald’Histoire et d’Art.

[61] Leplongeon A, Goring-Morris AN. Terminal Pleistocene lithic variability in the Western Negev (Israel): Is there any evidence for contacts with the Nile Valley? Journal of Lithic Studies. 2018;5(1):1–39. doi: 10.2218/jls.2614

[62] Sánchez-Martínez J, Roda Gilabert X, Vega Bolívar S, Martínez-Moreno J, Benito-Calvo A, Mora Torcal R. Beyond Shapes: Core Reduction Strategies in the Magdalenian of Cova Gran de Santa Linya (NE Iberia). Journal of Paleolithic Archaeology. 2022;5(1):7. doi: 10.1007/s41982-022-00115-x

[63] Delpiano D, Peresani M, Pastoors A. The contribution of 3D visual technology to the study of Palaeolithic knapped stones based on refitting. Digital Applications in Archaeology and Cultural Heritage. 2017;4:28–38. doi: 10.1016/j.daach.2017.02.002

[64] Magnani M. Three-dimensional alternatives to lithic illustration. Advances in Archaeological Practice. 2014;2(4):285–97. doi: 10.7183/2326-3768.2.4.285

[65] Magnani M, Douglass M, Schroder W, Reeves J, Braun DR. The digital revolution to come: Photogrammetry in archaeological practice. American Antiquity. 2020;85(4):737–60. doi: 10.1017/aaq.2020.59

[66] Abel RL, Parfitt S, Ashton N, Lewis SG, Scott B, Stringer C. Digital preservation and dissemination of ancient lithic technology with modern micro-CT. Computers & Graphics. 2011;35(4):878–84. doi: 10.1016/j.cag.2011.03.001

[67] Grosman L, Smikt O, Smilansky U. On the application of 3-D scanning technology for the documentation and typology of lithic artifacts. Journal of Archaeological Science. 2008;35(12):3101–10. doi: 10.1016/j.jas.2008.06.011

[68] Shott MJ, Trail BW. Exploring new approaches to lithic analysis: laser scanning and geometric morphometrics. Lithic Technology. 2010;35(2):195–220. doi: 10.1080/01977261.2010.11721090

[69] Barone S, Neri P, Paoli A, Razionale AV. Automatic technical documentation of lithic artefacts by digital techniques. Digital Applications in Archaeology and Cultural Heritage. 2018;11:e00087. doi: 10.1016/j.daach.2018.e00087

[70] Polo M-E, Felicísimo Á. Analysis of uncertainty and repeatability of a low-cost 3D laser scanner. Sensors. 2012;12(7):9046–54. doi: 10.3390/s120709046 23012532 PMC3444090

[71] Marcy AE, Fruciano C, Phillips MJ, Mardon K, Weisbecker V. Low resolution scans can provide a sufficiently accurate, cost-and time-effective alternative to high resolution scans for 3D shape analyses. PeerJ. 2018;6:e5032. doi: 10.7717/peerj.5032 29942695 PMC6016532

[72] Porter ST, Roussel M, Soressi M. A simple photogrammetry rig for the reliable creation of 3D artifact models in the field: lithic examples from the Early Upper Paleolithic sequence of Les Cottés (France). Advances in Archaeological Practice. 2016;4(1):71–86. doi: 10.7183/2326-3768.4.1.71

[73] Nakazawa Y, Izuho M, Takakura J, Yamada S. Toward an understanding of technological variability in microblade assemblages in Hokkaido, Japan. Asian Perspectives. 2005:276–92. doi: 10.1353/asi.2005.0027

[74] Langlais M. Dynamiques culturelles des sociétés magdaléniennes dans leurs cadres environnementaux: Enquête sur 7000 ans d’évolution de leurs industries lithiques entre Rhône et Ebre. 2007. https://hal.science/tel-03097690.

[75] Brou L, Le Brun-Ricalens F. Burins carénés et busqués: des nucléus à lamelles. L’apport des remontages du gisement de Thèmes (Yonne). In: de Araújo Igreja M, Bracco J-P, Le Brun-Ricalens F, editors. Burins préhistoriques: formes, fonctionnements et fonctions: Musée National d’Histoire d’Art; 2006. p. 225–38.

[76] Morales JI, Lorenzo C, Vergès JM. Measuring retouch intensity in lithic tools: a new proposal using 3D scan data. Journal of Archaeological Method and Theory. 2015;22:543–58.

[77] Falcucci A, Peresani M. The contribution of integrated 3D model analysis to Protoaurignacian stone tool design. Plos One. 2022;17(5):e0268539. doi: 10.1371/journal.pone.0268539 35584150 PMC9116640

[78] Wyatt-Spratt S. After the revolution: a review of 3D modelling as a tool for stone artefact analysis. Journal of Computer Applications in Archaeology. 2022. doi: 10.5334/jcaa.103

[79] Duca D. The ecosystem of technologies for social science research, data. Zenodo. 2019. Epub 1.

[80] Zangrossi F, Delpiano D, Cocilova A, Ferrari F, Balzani M, Peresani M. 3D visual technology applied for the reconstruction of a Paleolithic workshop. Journal of Archaeological Science: Reports. 2019;28:102045. doi: 10.1016/j.jasrep.2019.102045

[81] Lombao D, Falcucci A, Moos E, Peresani M. Unravelling technological behaviors through core reduction intensity. The case of the early Protoaurignacian assemblage from Fumane Cave. Journal of Archaeological Science. 2023;160:105889. doi: 10.1016/j.jas.2023.105889

[82] Martínez-Moreno J, Mora R, de la Torre I. The Middle-to-upper Palaeolithic transition in Cova Gran (Catalunya, Spain) and the extinction of Neanderthals in the Iberian peninsula. Journal of Human Evolution. 2010;58(3):211–26. doi: 10.1016/j.jhevol.2009.09.002 20097404

[83] Polo-Díaz A, Benito-Calvo A, Martínez-Moreno J, Mora Torcal R. Formation processes and stratigraphic integrity of the Middle-to-Upper Palaeolithic sequence at Cova Gran de Santa Linya (southeastern Prepyrenees of Lleida, Iberian peninsula). Quaternary International. 2016;417:16–38. doi: 10.1016/j.quaint.2015.10.113

[84] Mora Torcal R, Martínez Moreno J, Roy Sunyer M, Benito-Calvo A, Polo-Díaz A, Samper Carro S. Contextual, technological and chronometric data from Cova Gran: their contribution to discussion of the Middle-to-Upper Paleolithic transition in Northeastern Iberia. Quaternary International 2018;473:30–43. doi: 10.1016/j.quaint.2016.05.017

[85] Samper Carro SC, Martínez-Moreno J, Mora R. Wind of change: zooarchaeological approach to the Middle–Upper Palaeolithic transition in Cova Gran of Santa Linya (Lleida, south-eastern Pre-Pyrenees). Journal of Paleolithic Archaeology. 2020;3(4):989–1031. doi: 10.1007/s41982-020-00066-1

[86] Tostevin GB. Levels of theory and social practice in the reduction sequence and chaîne opératoire methods of lithic analysis. PaleoAnthropology. 2011:351–75. doi: 10.4207/pa.2011.art64

[87] Roebroeks W. From find scatters to early hominid behaviour: a study of Middle Palaeolithic riverside settlements at Maastricht-Belvédère (The Netherlands): Analecta Praehistorica Leidensia; 1988.

[88] Roy Sunyer M. Materias primas líticas y su explotación durante la prehistoria en el prepirineo oriental (NE de Iberia). 2016. http://hdl.handle.net/10803/400712.

[89] Bon F. L’Aurignacien entre mer et océan: réflexion sur l’unité des phases anciennes de l’Aurignacien dans le Sud de la France: Société préhistorique française; 2002.

[90] Bon F. Little big tool. Enquete autour du succés de la lamelle. In: Le Brun-Ricalens F, J-G B, Bon F, editors. Productions lamellaires attribuées à l’Aurignacien Chaînes opératoires et perspectives technoculturelles, (actes 14e Congrès UISPP, Université de Liège, Belgique, 2–8 sept 2001, session 6). Luxemburg: Musée National d’Histoire et d’Art de Luxembourg; 2005. p. 479–84.

[91] Bon F, Teyssandier N, Bordes J-G. La signification culturelle des équipements lithiques. In: Otte M, editor. Les Aurignaciens. Paris: Éditions errance; 2010. p. 49–72.

[92] Falcucci A, Conard NJ, Peresani M. A critical assessment of the Protoaurignacian lithic technology at Fumane Cave and its implications for the definition of the earliest Aurignacian. PLoS One. 2017;12(12):e0189241. doi: 10.1371/journal.pone.0189241 29216284 PMC5720803

[93] Ortega Cobos D, Soler Masferrer N, Maroto Genover J. La prodution de lamelles pendant l’Aurignacien archaïque dans la grotte de l’Arbreda: organisation de la production, variabilité des méthodes et des objectifs. In: Le Brun-Ricalens F, Bordes J-G, Bon F, editors. Productions lamellaires attribuées à l’Aurignacien: chaînes opératiores et perspectives technoculturelles (Actes du XIVe congrès de l’UISPP, Université de Liège, 2001). Luxemburg: Musée National d’Histoire et d’Art de Luxemburg; 2005. p. 359–73.

[94] Teyssandier N. En route vers l’Ouest: les débuts de l’Aurignacien en Europe: John and Erica Hedges Limited; 2007.

[95] Sánchez-Martínez J, Calmet K, Martínez-Moreno J, Roda Gilabert X. ReViBE: protocol for Refit Visualisation of lithic reduction sequences using the Blender Engine. Protocolsio. 2024. doi: 10.17504/protocols.io.ewov1qxqkgr2/v2

[96] Le Brun-Ricalens F, Bordes J-G. Productions lamellaires attribuées à l’Aurignacien: chaînes opératoires et perspectives technoculturelles: actes du XIVe congrès de l’UISPP, Université de Liège, 2–8 septembre 2001, session 6: paléolithique supérieur, colloque C6. 7: Musée national d’histoire et d’art; 2005.

[97] Grün A, Remondino F, Zhang L. Photogrammetric reconstruction of the great Buddha of Bamiyan, Afghanistan. The Photogrammetric Record. 2004;19(107):177–99. doi: 10.1111/j.0031-868X.2004.00278.x

[98] Scopigno R, Callieri M, Cignoni P, Corsini M, Dellepiane M, Ponchio F, et al. 3D models for cultural heritage: Beyond plain visualization. Computer. 2011;44(7):48–55. doi: 10.1109/MC.2011.196

[99] Bruno F, Bruno S, De Sensi G, Luchi M-L, Mancuso S, Muzzupappa M. From 3D reconstruction to virtual reality: A complete methodology for digital archaeological exhibition. Journal of Cultural Heritage. 2010;11(1):42–9. doi: 10.1016/j.culher.2009.02.006

[100] De Reu J, Plets G, Verhoeven G, De Smedt P, Bats M, Cherretté B, et al. Towards a three-dimensional cost-effective registration of the archaeological heritage. Journal of Archaeological Science. 2013;40(2):1108–21. doi: 10.1016/j.jas.2012.08.040

[101] Milosz M, Kęsik J. Special Issue on 3D Information Technologies in Cultural Heritage Preservation and Popularization—Motivations, Works Overview, and the Future. Applied Sciences. 2022;13(1):204. doi: 10.3390/app13010204

[102] Vicente-Saez R, Martínez-Fuentes C. Open Science now: A systematic literature review for an integrated definition. Journal of Business Research. 2018;88:428–36. doi: 10.1016/j.jbusres.2017.12.043

[103] Wilkinson MD, Dumontier M, Aalbersberg IJ, Appleton G, Axton M, Baak A, et al. The FAIR Guiding Principles for scientific data management and stewardship. Scientific Data. 2016;3(1):160018. doi: 10.1038/sdata.2016.18 26978244 PMC4792175

[104] Nicholson C, Kansa S, Gupta N, Fernandez R. Will It ever be FAIR?: Making archaeological data findable, accessible, interoperable, and reusable. Advances in Archaeological Practice. 2023;11(1):63–75. doi: 10.1017/aap.2022.40

[105] Munafò MR, Nosek BA, Bishop DV, Button KS, Chambers CD, Percie du Sert N, et al. A manifesto for reproducible science. Nature human behaviour. 2017;1(1):1–9. doi: 10.1038/s41562-016-0021 33954258 PMC7610724

[106] Marwick B. Computational reproducibility in archaeological research: Basic principles and a case study of their implementation. Journal of Archaeological Method and Theory. 2017;24(2):424–50. doi: 10.1007/s10816-015-9272-9

[107] Sánchez-Martínez J, Calmet K, Martínez Moreno J, Roda Gilabert X. ReViBe overview videos <https://ddd.uab.cat/record/287507>. 2023.

[108] Sánchez-Martínez J, Calmet K, Martínez-Moreno J, Roda Gilabert X. Replication Data for ReViBE: protocol for Refit Visualisation of lithic reduction sequences using the Blender Engine. In: Universitat Autònoma B, editor. V2 ed: CORA.Repositori de Dades de Recerca; 2024.

