# Supplementary material for: Virtual reconstruction of stone tool refittings by using 3D modelling and the Blender Engine: The application of the “ReViBE” protocol to the archaeological record
Source: PLoS One. 2024 Aug 29;19(8):e0309611. doi: 10.1371/journal.pone.0309611 (PMC11361422; doi:10.1371/journal.pone.0309611)
Supplement: S1 File — The references to the protocol on this article are expressed as (S1 Part X in S1 File). All the raw data needed to replicate this protocol has been uploaded in the Research Data Repository (CORA) (https://doi.org/10.34810/data924), including the pictures taken and used on this protocol (.jpg), the three dimensional model of each refitted artefact (.mtl and.obj), an interactive 3D model of the refitted sequence (3D.pdf) the flowchart of the refitted sequence, the table with technical attributes [108]. A step-by-step explanatory video of this protocol is available in the digital deposit of documents of Universitat Autònoma de Barcelona [107]. (PDF) [file pone.0309611.s001.pdf]

Apr 10, 2024 Version 2

ReViBE: protocol for Refit Visualisation of lithic reduction sequences using the Blender Engine V.2

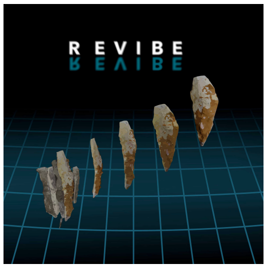

DOI
dx.doi.org/10.17504/protocols.io.ewov1qxqkgr2/v2

Javier Sánchez-Martínez[1,2], Katia Calmet[1], Jorge Martínez-Moreno[1], Xavier Roda Gilabert[1,3]

[1]CEPAP-UAB - Centre d'Estudis del Patrimoni Arqueològic de la Prehistòria, Universitat Autònoma de Barcelona, 08193 Bellaterra, Spain;
[2]ICArEHB – The interdisciplinary Center for Archaeology and Evolution of Human Behaviour, Universidade do Algarve, Campus de Gambelas, 8005-139 Faro, Portugal;
[3]CASEs- Culture, Archaeology, and Socio-Ecological Dynamics group. Universitat Pompeu Fabra. Departament d'Humanitats. 08005 Barcelona, Spain.

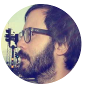

Xavier Roda Gilabert
CASEs- Culture, Archaeology, and Socio-Ecological Dynamics g...

OPEN ACCESS

DOI: **dx.doi.org/10.17504/protocols.io.ewov1qxqkgr2/v2**

Protocol Citation: Javier Sánchez-Martínez, Katia Calmet, Jorge Martínez-Moreno, Xavier Roda Gilabert 2024. ReViBE: protocol for Refit Visualisation of lithic reduction sequences using the Blender Engine. **protocols.io**
https://dx.doi.org/10.17504/protocols.io.ewov1qxqkgr2/v2 Version created by **Xavier Roda Gilabert**

Manuscript citation:

Sánchez-Martínez J, Calmet, K, Martínez Moreno J, Roda Gilabert X (submited) Virtual reconstruction of stone tool refitting by using 3D modelling and the Blender Engine: the application of the “ReViBE” protocol to the archaeological record. PLOS ONE

License: This is an open access protocol distributed under the terms of the **Creative Commons Attribution License**, which permits unrestricted use, distribution, and reproduction in any medium, provided the original author and source are credited

Protocol status: Working
We use this protocol and it's working

Created: April 10, 2024

Last Modified: April 10, 2024

Protocol Integer ID: 98019

Keywords: material culture, stone tools, lithic refittings, photogrammetry, Blender, 3D modelling

Funders Acknowledgement:
Program for the Requalification of the University System Margarita Salas
Grant ID: CA1/RSUE/2022-889183
Program for the Requalification of the University System Margarita Salas
Grant ID: CA1/RSUE/2021-707703
Human occupation during the Upper Pleistocene and Holocene in the south-eastern Pyrenees
Grant ID: PID2022-136363NB-I00
National Public Employment Service
Grant ID: NextGenerationEU

Abstract

Here, we introduce ReViBE, a step-by-step protocol for visualising lithic refits through the utilisation of imaging technologies, photogrammetry, 3D modelling and Blender animation software. Lithic refittings represent high-resolution temporal sequences that shed light in cognitive, organisational and taphonomic aspects of the archaeological record, and have remained integral for the archaeological science since its inception. Despite their popularity, little work has been focused on the representation of refittings, traditionally undertaken through photography and archaeological drawing. While the use of these recording tools are widespread in the discipline, their application in refittings has limitations, as they fail to capture the three-dimensional nature of lithic reduction.

Our ReViBE protocol addresses this issue through a step-by-step procedure that combines the creation of 3D models in refittings and their animation, allowing a more dynamic visualization of lithic reduction sequences in high definition. The protocol consists of three main phases in which we explain how to perform photogrammetry on the refitted artifacts, create three-dimensional models from the captured images (e.g., Photoscan/Metashape), and finally, generate animation by reconstructing the motion of each individual piece in a temporal sequence (e.g., Blender).

Furthermore, we suggest incorporating protocols employing 3D modelling to aid in the digital visualization of material culture, enhancing the accessibility and study of archaeological heritage.

Content:

Part 1 – Initial remarks and set up preparation

Part 2 – Obtention of 3D models using photogrammetry

Part 3 – Converting images to 3D

Part 4 – Creation of animation sequences using Blender

Supplementary video

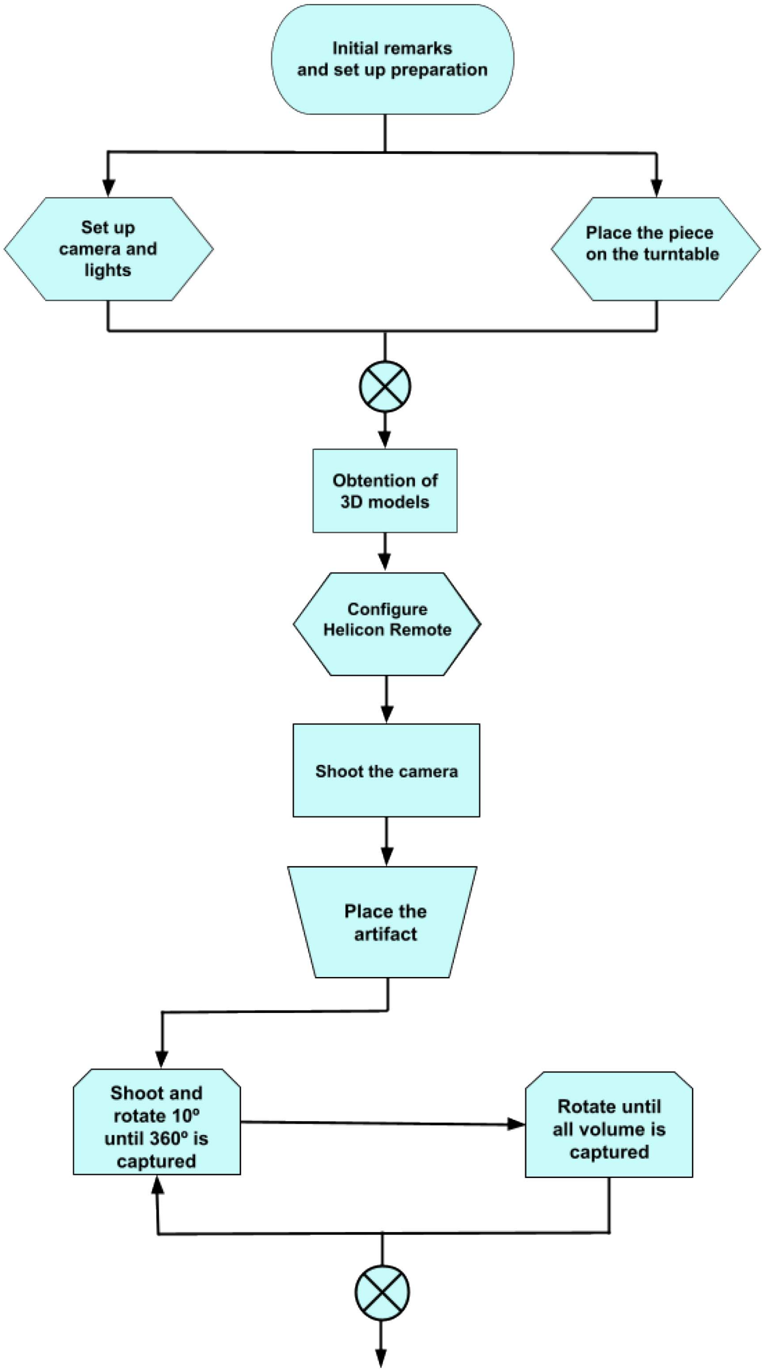
Initial remarks and set up preparation
Set up camera and lights
Place the piece on the turntable
Obtention of 3D models
Configure Helicon Remote
Shoot the camera
Place the artifact
Shoot and rotate 10º until 360º is captured
Rotate until all volume is captured

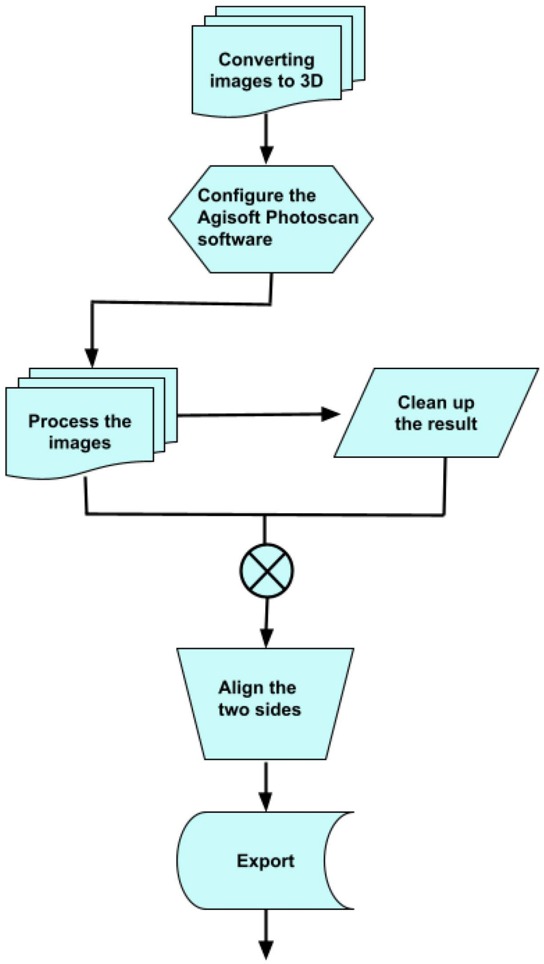
Converting images to 3D
Configure the Agisoft Photoscan software
Process the images
Clean up the result
Align the two sides
Export

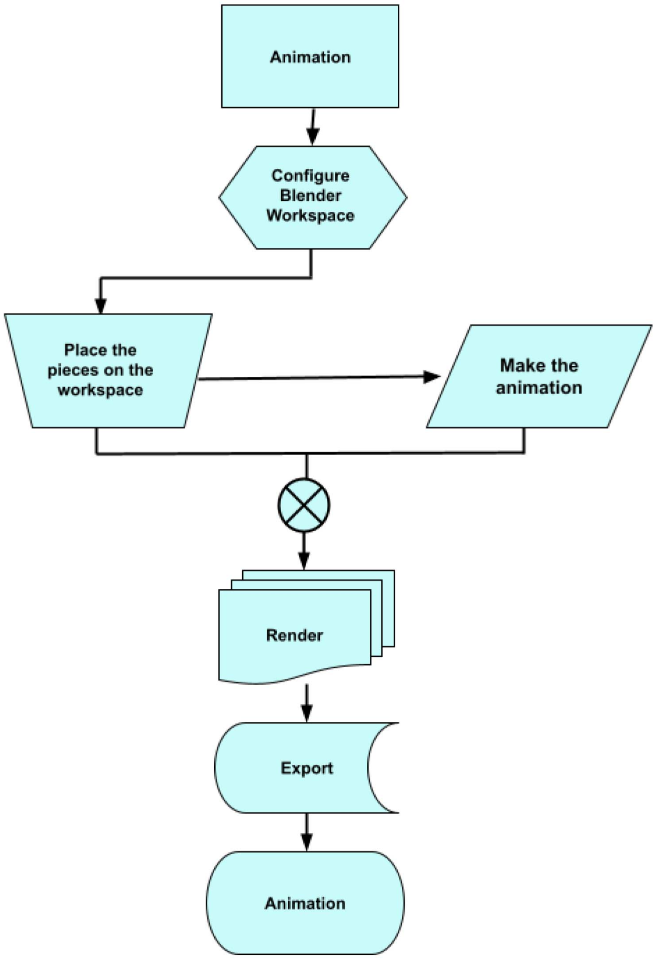

Flowchart showing the workflow when using the ReViBe protocol according to the standardised symbols of ISO 5807:1985 and Myler, 1998.

Image Attribution

All images by Katia Calmet and CEPAP-UAB.

Guidelines

Typographical emphasis used in the protocol:

- References to materials and software used in *italics*, e.g. *Helicon Focus*
- Software features in **bold**, e.g. **Align Photos**
- Computational workflows linked by angle brackets (>), e.g. Workflow > Build Mesh
- Keyboard buttons indicated by angle markers (< >), e.g. <Right-Click >

This protocol was developed using Helicon Remote© 4.4.4, Agisoft Photoscan© 1.5.2 and Blender© 3.6. Different versions of the software will present slightly differing interfaces, but the general workflow is still the same.

The last section of the protocol contains a video with an extended overview of the whole process.

Materials

Image capturing:

- Camera (ideally with 35mm - 80mm focal length)
- Tripod
- Light box (optional)
- PC or laptop
- Rotating turntable
- Modelling clay and plastic wrap
- Photogrammetric scale marker

3D scanning and processing software:

- Helicon Remote
- Agisoft PhotoScan
- Blender

Safety warnings

Application of this protocol is risk-free

Before start

Note

According to the size of the pieces, the preparation will vary in terms of the distance from the camera to the objects.

To obtain the 3D model we can use photogrammetry or 3D scanning.

Part 1 - Initial remarks and set up preparation

1 Place the *camera* on a *tripod* (ideally with a zoom lens with a focal length between 35mm and 80mm). Do not use an autofocus lens.

2 Place the artefact in a *light box* or, if this is not available, on a plain coloured background.

3 Place the artefact on a *turntable* within the workspace.

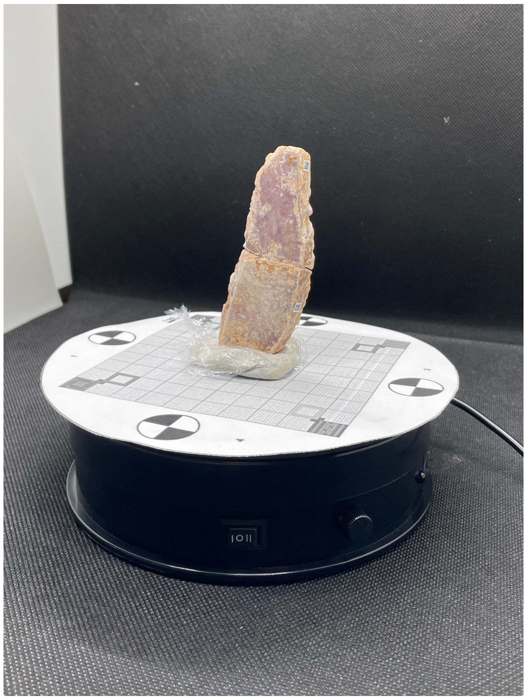

Fig. 1: Artefact on the *turntable* inside the light box.

3.1 **Note**

The artefact must always be in the center of the frame

3.2 Use a *Photogrammetric scale marker* to scale the images afterwards. Three free versions of the photogrammetric scale can be downloaded at this **link** (Porter, Roussel & Soressi, 2016a; Porter, Roussel & Soressi, 2016b).

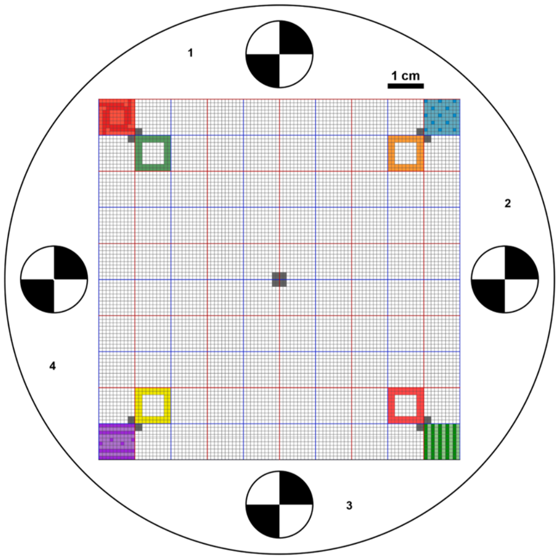

Fig. 2: Photogrammetric scale marker.

3.3 If the artefact will not stand due to its irregular shape, use an appropriate amount of *modelling clay* wrapped in plastic wrap to create a modifiable support surface.

4 With 3 light sources, create diffused lighting from both sides and above. Try to avoid large shadows on the background surface and on the piece.

5 Shoot with the camera connected to a laptop running *Helicon Remote©* or any other remote software.

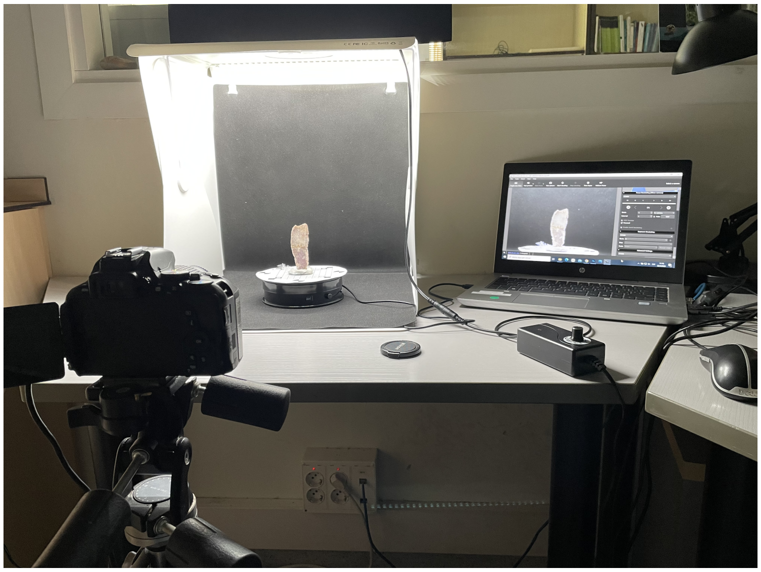

Fig. 3: Set up with the *camera* at a front angle, connected to the *Helicon Remote.*

Part 2 - Obtention of 3D models using photogrammetry

6 Open *Helicon Remote* .

7 <Click> **Toggle Live view** for seeing what is on the frame.

8 **Configure Helicon Remote Software**

Set the camera on the **Camera settings** following the next parameters:

F- number between 2 and 1.3
Aperture between 8 and 14
100 ISO

9 <Click> on **Take picture** on the top toolbar.

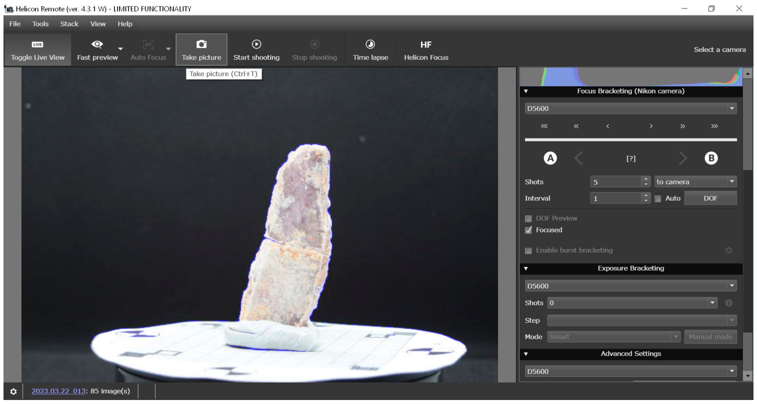

Fig. 4: *Helicon Remote* interface with **Live View** image activated.

10 Move the artefact 10 degrees on the turntable.

11 Take another picture.

12 Repeat steps , , to capture all the 360º.

13 Change the position of the camera to a high-angle.

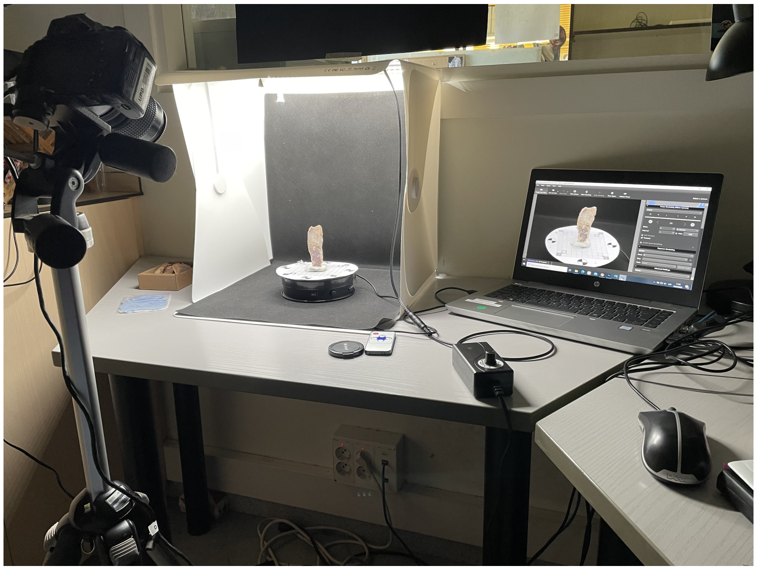

Fig. 5: Set up with the camera at a high-angle.

14 Repeat steps , , from this angle.

15 Return the camera to a frontal angle and turn the artefact upside down.

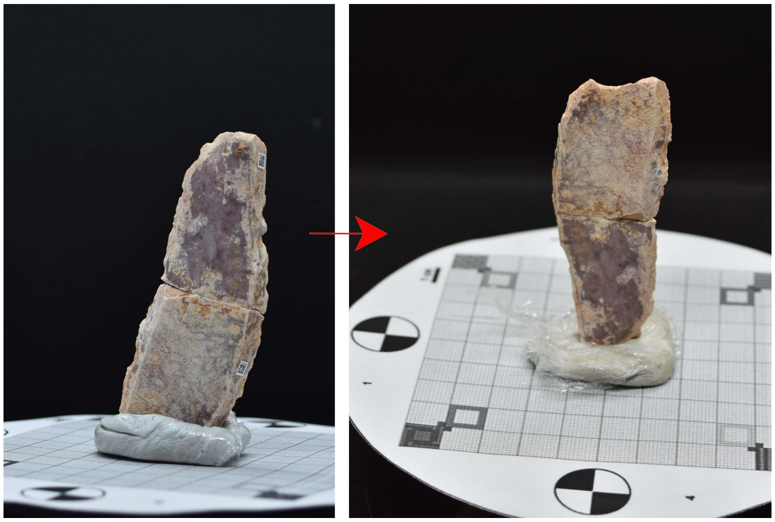

Fig. 6: Change of the position of the artefact (180º).

16 Repeat *steps* , , with the artefact upside down.

17 Repeat *steps* *and* .

Part 3 - Converting images to 3D

18 **Opening and configuring the *Agisoft Photoscan©* Software**

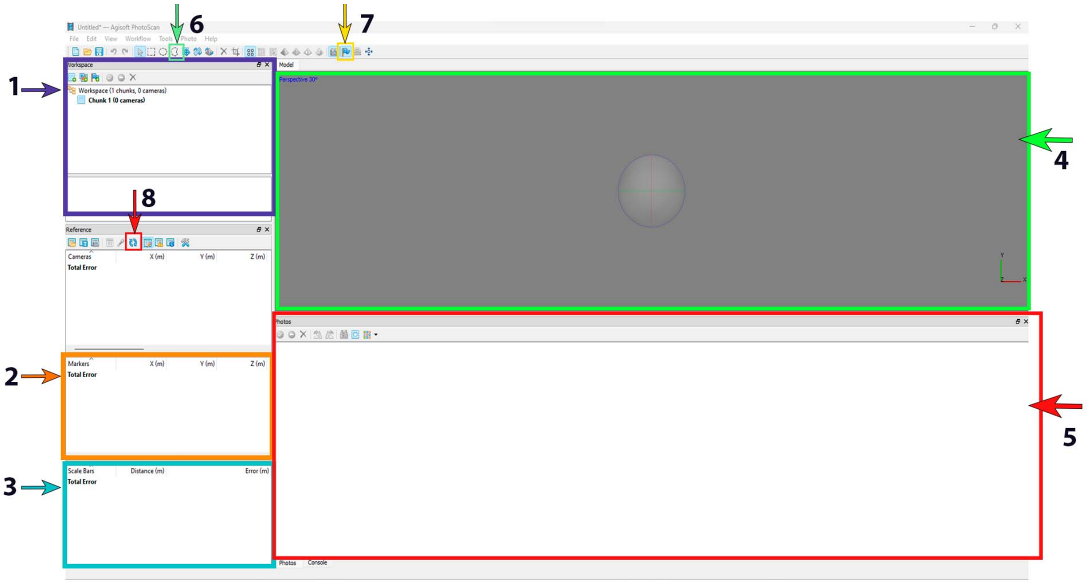

Fig. 7: Programme overview of the tools and panels used in ***Photoscan*** to transform the images into a 3D model.

Legend:

1. Workspace, to organise.
2. Markers to scale the artefact.
3. Scale bars to introduce the scale interval.
4. Model view, to see the development of the artefact.
5. Photo section where all the photos are separated. Masks can be applied in this area.
6. Freeform selection tool.
7. Markers.
8. Update, to apply the measurements to the scale bars.

In the left part of the screen, go to **Workspace** and tap <Right-click> on the mouse > **Add Chunk.**

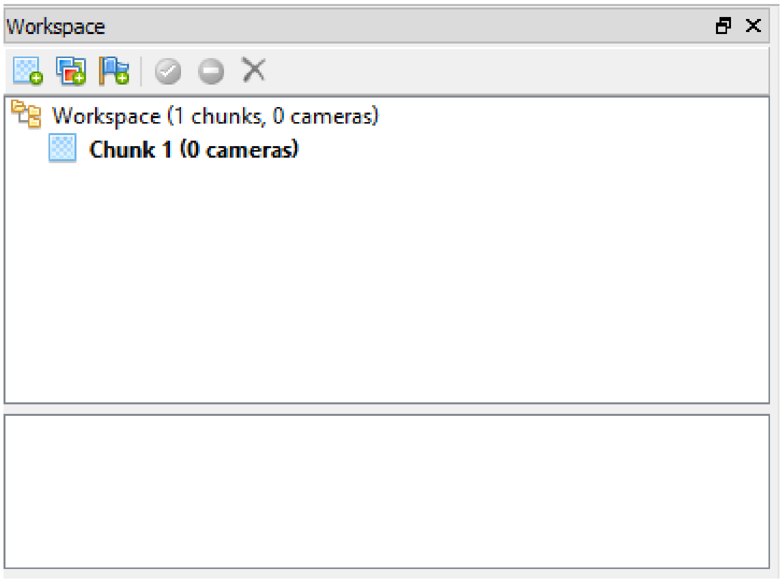

Fig. 8: Chunks are like folders where the photos are stored.

19 Go to the pictures folder on your computer and drop all the photos with the artefact in a single position, with the front angle and the high angle.

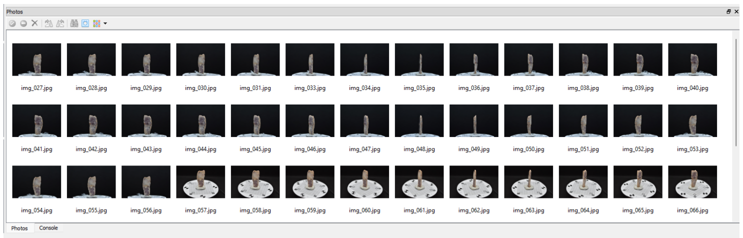

Fig. 9: Photos section of the *Photoscan* interface.

20 **Create a mask.**

Go to the **Photos section** and open one. Use the **magic wand** to select the background. <Right-click> and select **Add Selection**. Do this for each photo.

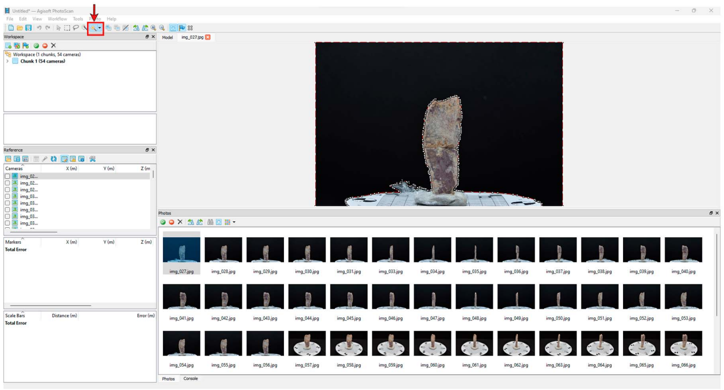

Fig. 10: The **magic wand** tool appears on the toolbar when you open an image.

21 Return to the model view.

22 Select **Workflow** on the toolbar and <Click> on **Align photos**.

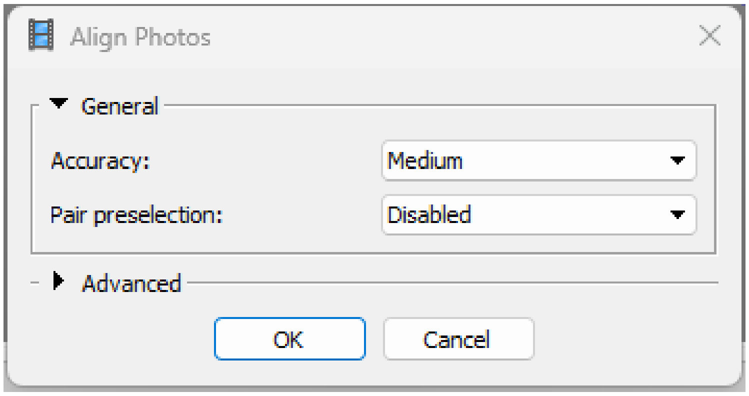

Fig. 11: Pop-up window, **Align Photos** preferences.

22.1

Note

If the alignment of the photos is not correct, try the alignment again.

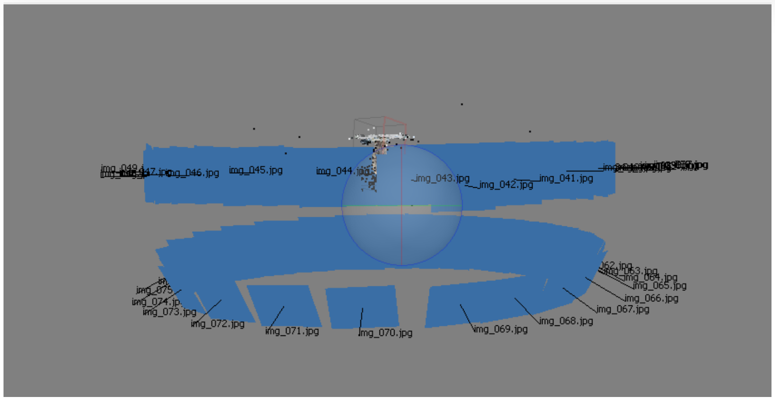

Fig. 12: The alignment works well if all the images appear to be wrapped around the artefact.

23 In the pop-up window, select **Accuracy > Medium** and **Pair preselection > Disabled**.

24 Select **Workflow** on the toolbar again and <Click> on **Build dense cloud**.

25 On the pop-up window select **Quality > Medium**.

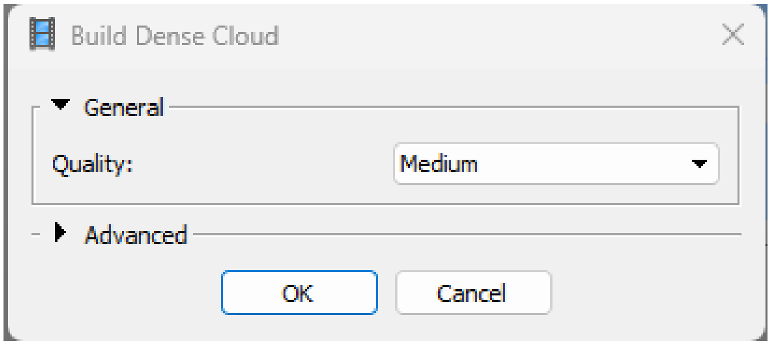

Fig. 13: Dense cloud setting.

26 Select **Workflow > Build Mesh**.

27 In the following pop-up window leave the default parameters and <Click> the **OK** button.

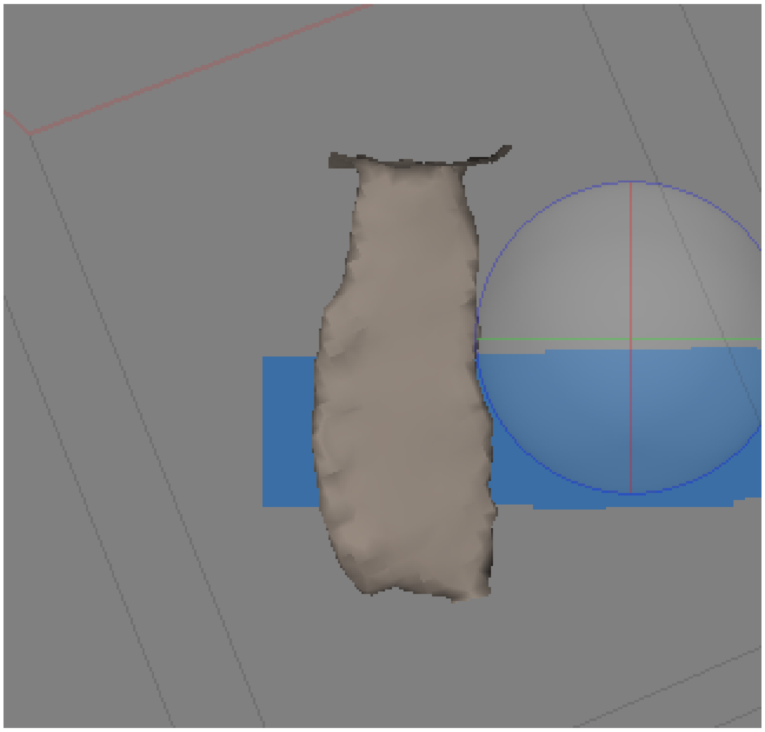

Fig. 14: Appearance of the artefact after creating the mesh.

28 Select **Workflow > Build Texture**.

29 In the following pop-up window leave the default parameters and<Click> the **OK** button.

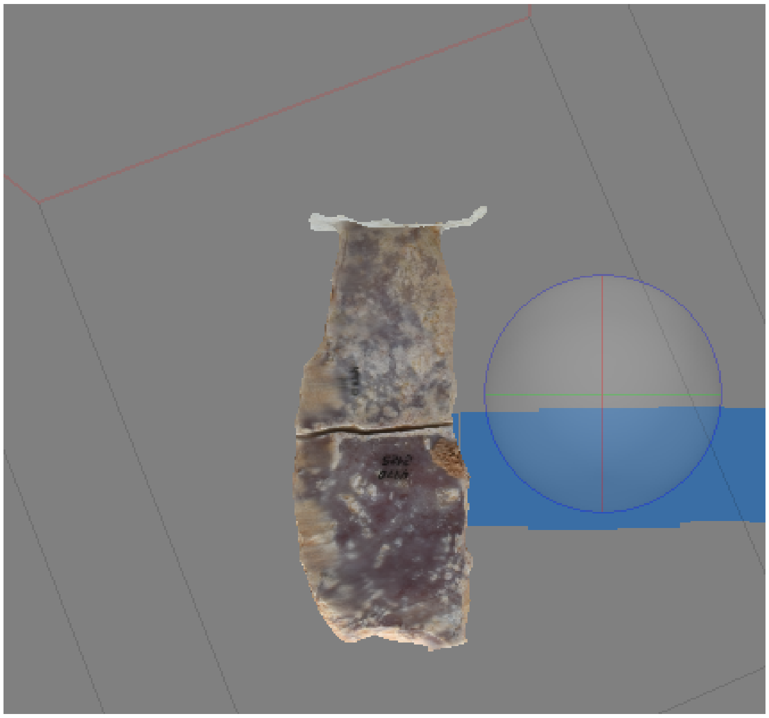

Fig. 15: Appearance of the artefact after creating the texture.

29.1 Note

If you cut a little above the clay, it will be easier to fit later.

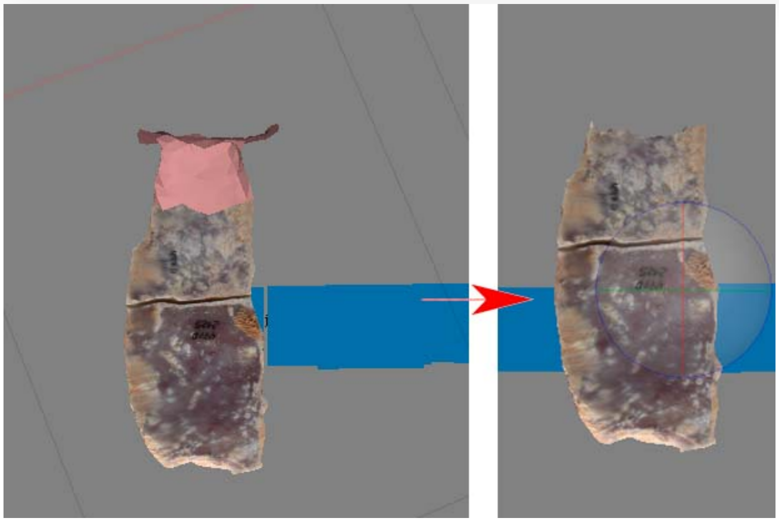

Fig. 16: Cutting up the artefact.

30 Clean up the artefact, use the **freeform selection tool** and draw over the parts that do not belong to the artefact, and press <DELETE> on your keyboard.

31 **Add Chunk 2** on the **Workspace.**

32 In the **Photos** panel, move all the photos that are left in the pictures folder with the artefact to the other position.

33 Repeat , , , , , .

34 Open **Chunk 1** and go to the **Photos** pane.

35 Open one of the photos from the high angle where the *Photogrammetric scale marker* is visible.

36 Using the **marker tool,** <Right-click> on one of the targets and select **Create Marker**.

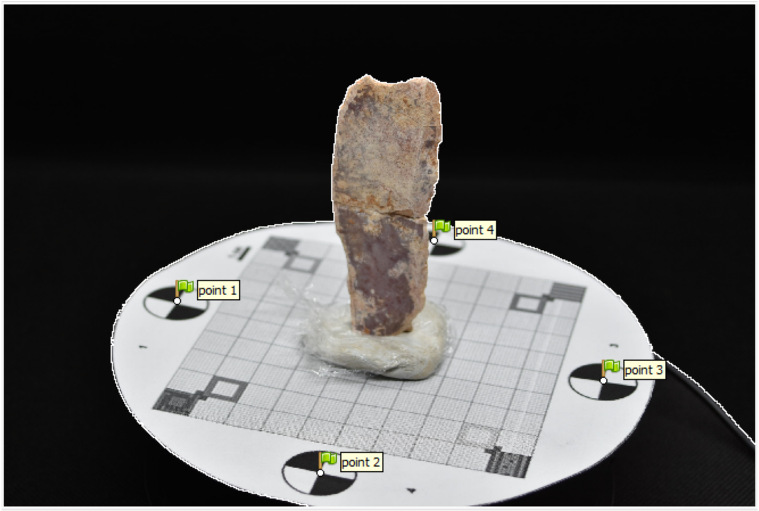

Fig. 17: The marker points use the *photogrammetric scale marker* as a reference.

37 Repeat on each reference target in the image.

<Right-click> on the **target > place marker >** choose the point that matches the one in the image above.

38 Repeat , in every high-angle photo.

39 On the **Reference** menu, go to the **Makers** section and select two points that are parallel to each other**,** <Right-click> and select **create scale bar**.

40 On the **scale bar,** enter the distance between the targets according to the distance in real life.

Note

Try not to cross the points.

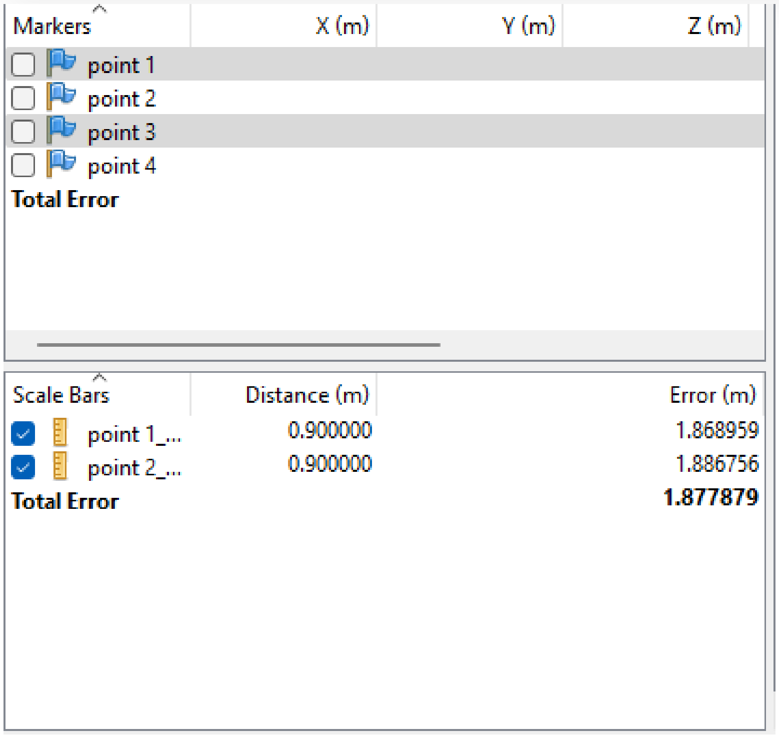

Fig. 18: Creating the scale bars, to scale the artefact.

41 <Click> the **Update** icon and return to the model.

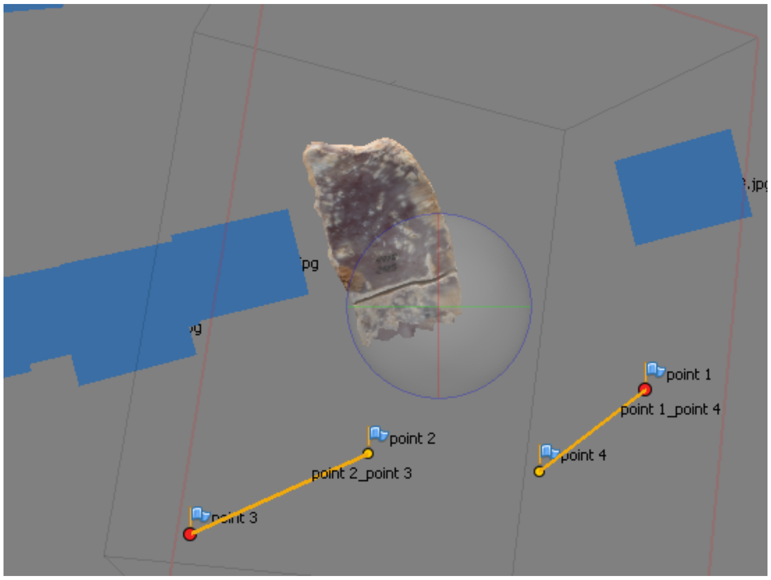

Fig. 19: Part of the scaled artefact.

42 Repeat , , , on chunk 2.

43 Now go to **Workflow > Align Chunks**.

44 In the pop-up window, select the two chunks and leave all the parameters as default.

45 **Workflow > merge chunks.**

46 In the pop-up window, select the two chunks and choose **Merge models.**

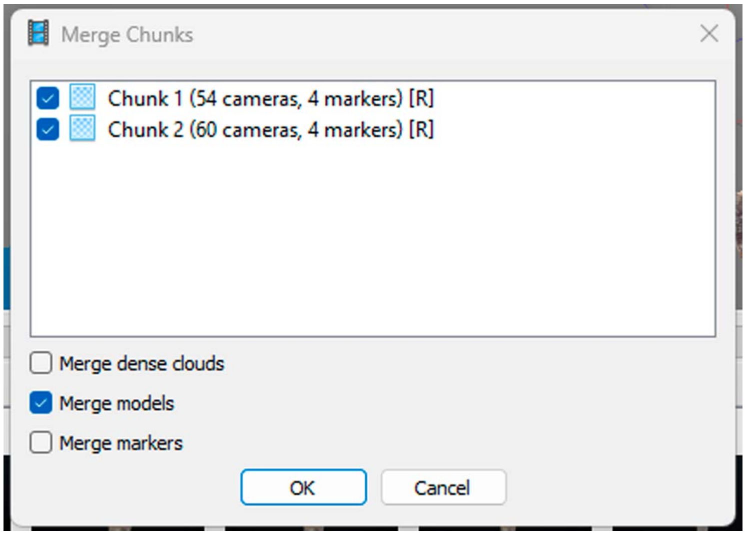

Fig. 20: Merge chunks window.

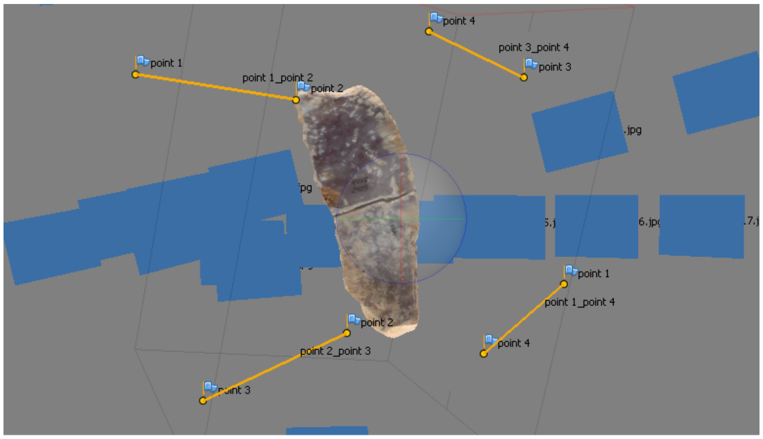

Fig. 21: Final result.

47 Export

File > Export Model > Export OBJ/FBX/KMZ...> OK

Part 4- Creating animated sequences using Blender

48 Blender is a flexible tool with many possibilities that allows the creation of animation sequences using the 3D models previously obtained by photogrammetry. Here we explain the different steps that should be followed up to create core reduction animation sequences.
Open Blender > Select the **General** option at the first menu.

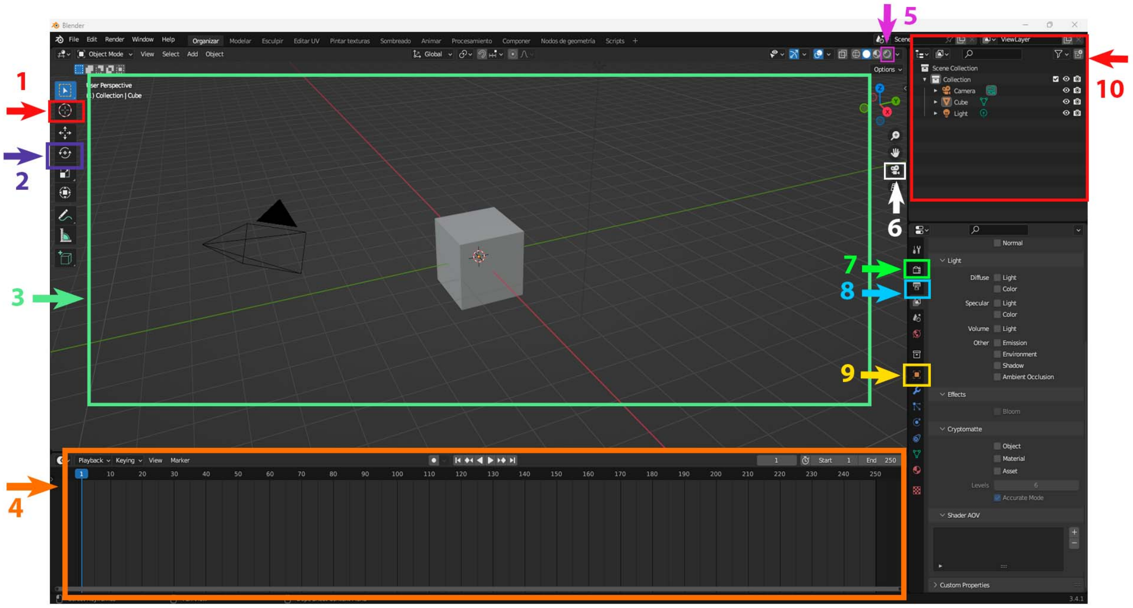

Fig. 22: Overview of the tools and panels that are used in Blender to animate the artefacts.

Legend:

1. 3D cursor (for moving objects arround the workspace).
2. Rotate tool (to rotate the object).
3. 3D view of the workspace.
4. Timeline (display of the movements in time by keyframes).
5. Viewport shading (view of the object with the texture).
6. Toggle the camera view.
7. Render Properties.
8. Output Properties.
9. Object Properties.
10. Scene collection (space with a list of all the objects).
11. Header (where the object mode options can be found).

49 Delete the cube by default, <Click> on the cube and press <DELETE>

50 **File > Import > Obj.** and browse for the object in the computer folder.

51 **Place all the artefacts on the workspace.**
<Click> on one of the artefacts.

51.1 **Note**

To move around the workspace, press the middle mouse button and drag. Use the mouse wheel to zoom in and out.

52 <Click> on the **viewport shading** to see the object with the texture.

53 Go to object on the **Header > Set Origin > Origin to centre of mass (surface).**

54 To move objects press <G> on the keyboard and move the mouse to position the object. To change the position more precisely go to the object properties. <Right-click> to set.

55 To rescale objects, <Click> on the object in the workspace, press<S> and move the mouse in the **object properties.**

56 To rotate the object, select the **rotate tool** and move the mouse on the axis you want to rotate or in the **object properties** panel.

57 Repeat *steps* , . . for each object one by one.

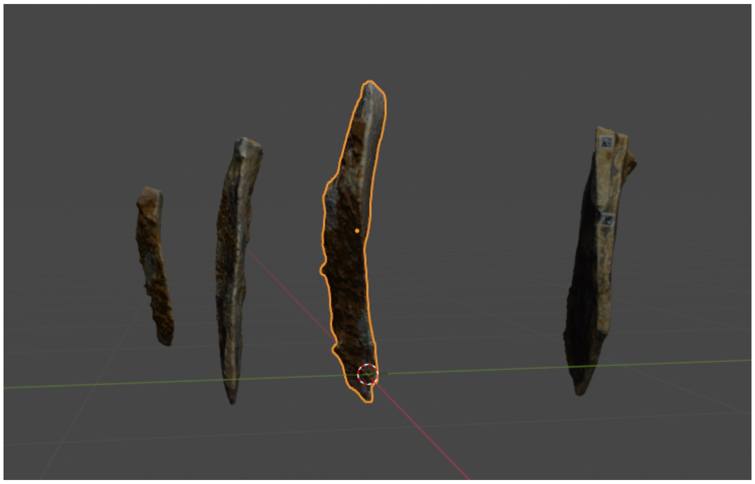

Fig. 23: Motion of objects on the **workspace**.

58 **Make the set up**
Go to the **Header > Add** and <Click> on **Camera.**

59 Select the camera by clicking above it and press <G> on the keyboard, move the mouse, and move the camera until you have the object in the frame.

60 <Click> on **Toggle the camera view** and see what's in the frame.

61 Adjust the frame, press <G>**,** and move the mouse, until you have the objects in the frame.

62 Go to the **Header > Add > Light > Point.**

63 Place **3 lights** and create a standard 3 point lighting, one light behind the object and two lights from the sides of the **camera**.

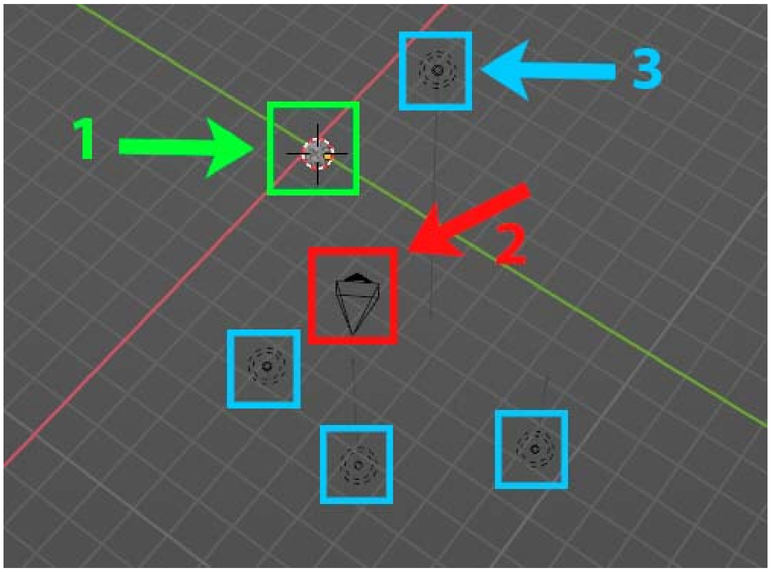

Fig. 24: Set configuration diagram.

Legend:
1. Artefacts/objects.
2. Camera.
3. Light point.

63.1 You can add another light to backfill.

64 **Animation**
Select **Auto Keying** on the **Timeline** to capture a keyframe every time an object moves.

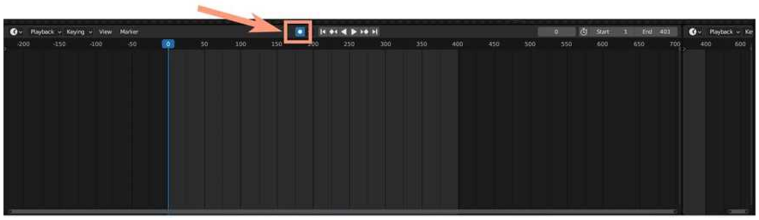

Fig. 25: Selecting **Auto Keying** on the **Timeline**.

65 Drag the bar on the **timeline** to the second you want the object's first action to end.

66 <Click> and drag the object you want to move to the first position.

67 Place the object where the object's action ends, then <Click> to fix it.

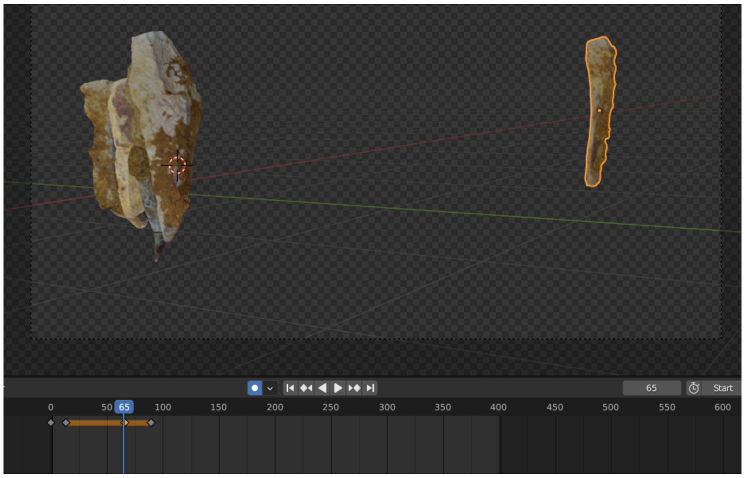

Fig. 26: Timeline view with keyframes per motion action.

68 Move the bar on the **timeline** to the second the action starts.

69 To rotate the object, set it to the second in which the object has not changed its position, move the bar on the **Timeline** to the second in which the rotation will end.

70 Drag the **3D cursor** over the object you want to rotate.

71 Use the **Rotate tool** and apply the rotation at the second the movement ends on the **Timeline**.

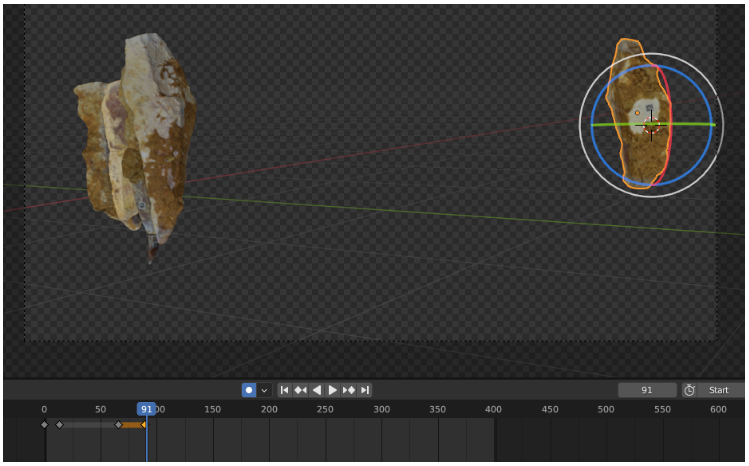

Fig. 27: Object rotation overview.

72 Repeat *steps* , , , for each object.

72.1

Note

Check all movements in the camera view and make sure the animation stays in the frame.

72.2

Note

To play the animation, tap the spacebar on your keyboard.

72.3

Note

Each object has its own timeline.

73 **Render**
Delimit the timeline where it says Start/End. Leave a few seconds at the end of the animation.

74 Go to the **Rendering** tab.

75 **Output properties > output >** name of the animation and where it's saved (folder) **> Format File > FFmpeg video > Colour profile RGB > Encoding > container > MPEG-4 > Video > H264 > Lossless > Good.**

76 **Render Properties > Scene > Render Engine** and change it to **Cycles**.

77 **Render Properties > Film > Transparent.**

78 Go to the **Render** tab (not rendering) **> render animation.**

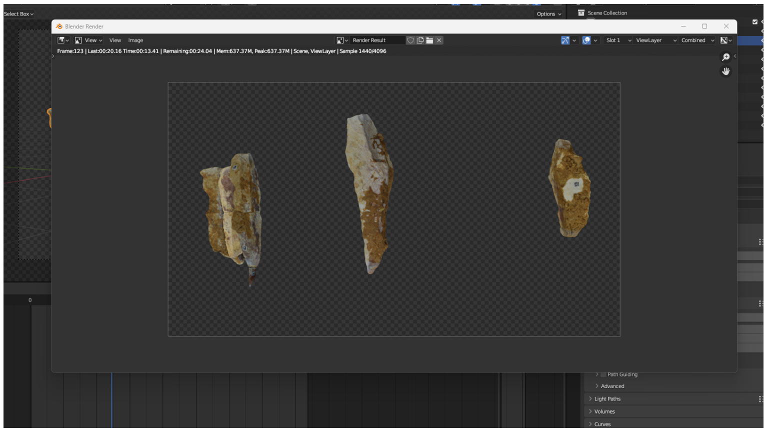

Fig. 28: Render window.

There are several export options for this software, depending on how you want to use it and how quickly you can reproduce it on your device. You can also capture still images for editing in other programs, such as Adobe Photoshop, or for uploading to a website.

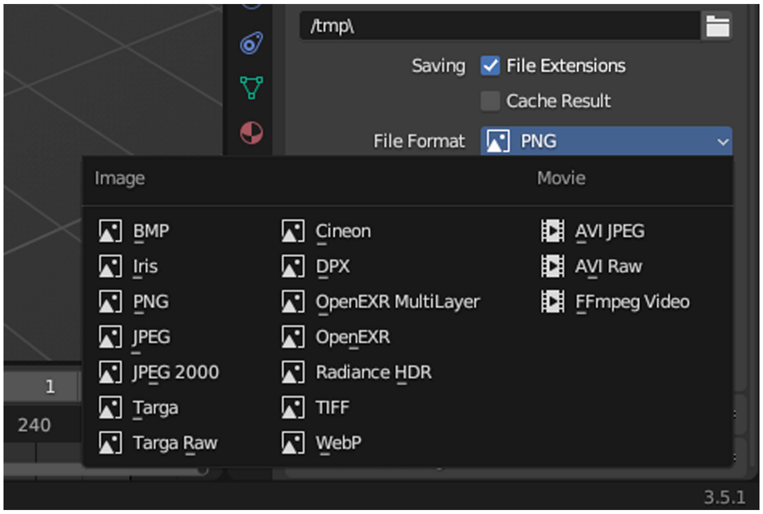

Fig. 29: Output options.

Supplementary video

79 The video included in this section shows a detailed step-by-step process following the procedure explained in this protocol. Sample materials to ensure reproducibility of the method are available from this link.

https://videosdigitals.uab.cat/almacen/downloads/998/18156.mp4

Protocol references

ISO 5807:1985 (1985) Information processing. Documentation symbols and conventions for data, program and system flowcharts, program network charts and system resources charts. International Organization for Standardization. February 1985, 25 p

Myler, HR (1998). *Fundamentals of engineering programming with C and Fortran*. Cambridge University Press.

Porter, ST; Roussel, M; Soressi, M. (2016a).A Simple Photogrammetry Rig for the Reliable Creation of 3D Artifact Models in the Field: Lithic Examples from the Early Upper Paleolithic Sequence of Les Cottés (France). etrieved from the Data Repository for the University of Minnesota, http://dx.doi.org/10.13020/D6201D.

Porter, ST, Roussel, M & Soressi, M (2016b). A simple photogrammetry rig for the reliable creation of 3D artefact models in the field: lithic examples from the Early Upper Paleolithic sequence of Les Cottés (France). Advances in Archaeological Practice, 4 (1), 71-86. https://doi.org/10.7183/2326-3768.4.1.71
